# Restoring Knee Flexor Strength Symmetry Requires 2 Years After ACL Reconstruction, But Does It Matter for Second ACL Injuries? A Systematic Review and Meta-analysis

**DOI:** 10.1186/s40798-023-00666-5

**Published:** 2024-01-05

**Authors:** Johan Högberg, Ramana Piussi, Johan Lövgren, Mathias Wernbom, Rebecca Simonsson, Kristian Samuelsson, Eric Hamrin Senorski

**Affiliations:** 1 Sportrehab Sports Medicine Clinic, Stampgatan 14, 411 01 Gothenburg, Sweden; 2 Sahlgrenska Sports Medicine Center, Gothenburg, Sweden; 3 Unit of Physiotherapy, Department of Health and Rehabilitation, Institute of Neuroscience and Physiology, Sahlgrenska Academy, University of Gothenburg, Box 455, 405 30 Gothenburg, Sweden; 4 Department of Orthopaedics, Institute of Clinical Sciences, Sahlgrenska Academy, University of Gothenburg, Gothenburg, Sweden; 5 The Rydberg Laboratory for Applied Sciences, Halmstad University, Box 823, 301 18 Halmstad, Sweden; 6 Active Physio Sports Medicine Clinic, Brogatan 23, 431 30 Gothenburg, Sweden; 7 Department of Orthopaedics, Sahlgrenska University Hospital, Mölndal, Sweden

**Keywords:** Anterior cruciate ligament, Hamstring tendon autograft, Limb symmetry index, Knee flexor strength, Knee flexor strength methodology, Second ACL injuries

## Abstract

**Background:**

It is unknown whether knee flexor strength recovers after anterior cruciate ligament (ACL) reconstruction with a hamstring tendon (HT) autograft and whether persistent knee flexor strength asymmetry is associated to a second ACL injury.

**Objective:**

We aimed to systematically review (1) whether knee flexor strength recovers after ACL reconstruction with HT autografts, and (2) whether it influences the association with a second ACL injury. A third aim was to summarize the methodology used to assess knee flexor strength.

**Design:**

Systematic review and meta-analysis reported according to PRISMA.

**Methods:**

A systematic search was performed using the Cochrane Library, Embase, Medline, PEDRo, and AMED databases from inception to December 2021 and until completion in January 2023. Human clinical trials written in English and conducted as randomized controlled trials, longitudinal cohort, cross-sectional, and case–control studies on patients with index ACL reconstructions with HT autografts harvested from the ipsilateral side were considered. Knee flexor strength was measured isokinetically in both the reconstructed and uninjured limb to enable the calculation of the limb symmetry index (LSI). The Risk of Bias Assessment Tool for Non-Randomized Studies was used to assess risk of bias for non-randomized studies and the revised Cochrane Risk of Bias tool was used for randomized controlled trials. For the meta-analysis, the LSI (mean ± standard error) for concentric knee flexor strength at angular velocities of 60°/second (s) and 180°/s preoperatively and at 3 months, 6 months, 12 months, and 24 months were pooled as weighted means with standard errors.

**Results:**

The search yielded 64 studies with a total of 8378 patients, which were included for the assessment of recovery of knee flexor strength LSI, and a total of 610 patients from four studies that investigated the association between knee flexor strength and second ACL injuries. At 1 year after ACL reconstruction, the knee flexor strength LSI had recovered to 89.0% (95% CI 87.3; 90.7%) and 88.3% (95% CI 85.5; 91.1%) for the velocities of 60°/s and 180°/s, respectively. At 2 years, the LSI was 91.7% (95% CI 90.8; 92.6%) and 91.2% (95% CI 88.1; 94.2%), for velocities of 60°/s and 180°/s, respectively. For the association between knee flexor strength and second ACL injuries, there was insufficient and contradictory data.

**Conclusions:**

There was low to very low certainty of evidence indicating that the recovery of knee flexor strength LSI, defined as ≥ 90% of the uninjured side, takes up to 2 years after ACL reconstruction with HT autografts. Whether knee flexor strength deficits influence the association of second ACL injuries is still uncertain. There was considerable heterogeneity in the methodology used for knee flexor strength assessment, which together with the low to very low certainty of evidence, warrants further caution in the interpretation of our results.

*Registration number:* CRD42022286773.

**Supplementary Information:**

The online version contains supplementary material available at 10.1186/s40798-023-00666-5.

## Key Points

- Achieving knee flexor strength limb symmetry requires up to 2 years in patients who have undergone ACL reconstruction with a HT autograft.
- The association between knee flexor strength asymmetry and the occurrence of second ACL injuries is largely unknown due to insufficient and contradictory data.
- There is great heterogeneity and a lack of reporting of the methodology when assessing knee flexor strength after ACL reconstruction.

**Supplementary Information:**

The online version contains supplementary material available at 10.1186/s40798-023-00666-5.

## Background

Recovering muscle strength in the knee extensors and flexors after anterior cruciate ligament (ACL) reconstruction is considered important as it helps to increase the likelihood of returning to knee-demanding activities [1, 2]. The knee extensors provide shock absorption and are responsible for controlling knee flexion, while the knee flexors are thought to limit excessive anterior tibial translation and provide rotational stability of the knee joint [3]. Consequently, the recovery of knee extension and flexion strength is regarded as a cornerstone during the rehabilitation process after an ACL injury, where the strength relative to the uninjured side expressed as a percentage [limb symmetry index (LSI)] is commonly used as guidance [4]. An expert consensus statement suggested a cut-off value of ≥ 90% in the LSI for the knee extensors and flexors as a proxy for a “successful” outcome [4]. While achieving an LSI of ≥ 90% for knee extension strength might reduce the risk of a second ACL injury after returning to sports [5–7], the question of whether a similar relationship exists for knee flexor strength has not been well studied.

A standardized assessment of knee flexor strength after ACL reconstruction is the first step in investigating the possible relevance of knee flexor strength symmetry for secondary ACL injuries. The knee flexors are a biarticular muscle group with functions over both the hip and the knee joints, which influence the interpretation of knee flexor strength assessments [8]. There are several aspects to consider with regard to the measurement of knee flexor strength, e.g., knee flexor peak torque has been reported to be lower at greater angles of knee flexion [9] and at more extended hip angles, such as in a prone or a supine position compared with a seated position [10–12]. Knee flexor strength may also be affected by the choice of autograft, with hamstring tendon (HT) autografts causing greater knee flexor strength deficits than both patellar tendon autografts and quadriceps tendon autografts [13, 14].

Collectively, knee flexor strength results are influenced by both the methodology for measuring knee flexor strength and the choice of autograft. In addition, there is a lack of knowledge relating to the role played by knee flexor strength for second ACL injuries.

The aim of this systematic review was therefore to summarize the available evidence regarding (1) the recovery of knee flexor strength, (2) the association between knee flexor strength and second ACL injuries, and (3) the methodology used when assessing knee flexor strength in patients after ACL reconstruction treated with an HT autograft.

## Methods

The Preferred Reporting Items for Systematic Review and Meta-Analyses (PRISMA) statement [15] was followed when reporting the present systematic review. This review was prospectively registered in the International Prospective Register of Systematic Reviews (PROSPERO) with registration ID CRD42022286773.

### Eligibility Criteria

The eligibility criteria were as follows: (1) studies written in English; (2) original clinical human studies designed as randomized controlled trials (RCT), prospective or retrospective longitudinal cohort studies, cross-sectional studies, and case–control studies; (3) studies presenting data on the LSI (mean ± standard deviation) for isokinetically measured knee flexor strength; and (4) studies investigating index ACL reconstructions with HT autografts harvested from the ipsilateral side. For the second aim, we included studies presenting data on knee flexor strength before second ACL injuries, after index ACL reconstruction with an HT autograft harvested from the ipsilateral side. Studies were excluded if (1) the entire population was < 16 years old, due to strength differences between the pediatric and adult populations [16]; (2) they solely investigated the effects of passive treatment (e.g., immobilization) or femoral nerve blockade on knee flexor strength, which could act as a confounding factor; (3) separate data for HT grafts could not be extracted due to reporting together with other graft types; and (4) the full text was not accessible. Furthermore, studies presenting LSI data for subgroups such as anterior knee pain and the incomplete regeneration of the HT were excluded, as well as literature reviews, systematic reviews, meta-analyses, case studies, conference abstracts, chapters from textbooks, opinion pieces, and editorials.

### Information Sources and Search Strategy

The literature search was performed by a medical librarian with expertise in electronic searches at the Biomedical Library at the University of Gothenburg, Gothenburg, Sweden. A systematic search was performed in December 2021 and a second search for the second aim in January 2022 using the Cochrane Library, Embase, Medline, PEDRo, and AMED databases. An updated systematic search was performed in January 2023 to include any recently published studies. A similar search strategy was used with adaptation to each database configuration (Additional file 1: The Search Strategy, Online resources 1–3). The search combined the use of medical subject headings (MeSH) with free-text terms including ACL, ACL reconstruction, semitendinosus, hamstring tendon autograft, harvest, knee flexor strength, muscle strength assessment, second ACL injury, subsequent, second, and synonyms. In addition, the reference lists of the included studies were screened for potential studies not previously identified.

### Selection Process

Two authors (JH and JL) independently reviewed all the titles and abstracts to determine eligibility. Studies deemed as eligible were then read in full text before potential inclusion. Any disagreement between the two authors was resolved by a discussion with the senior author (EHS). Data consisting of title, author, year of publication, journal, study type, purpose, conclusion, sample size, patient sex, patient age, sport, activity level, graft type, patients lost to follow-up, test equipment, contraction mode, test position, knee angle, angular velocity, timepoint of assessment after ACL reconstruction, relative strength and/or absolute strength, time of return to sport (RTS), time of injury after RTS, ipsilateral ACL injuries, contralateral ACL injuries, and other injuries were extracted into Excel (version 16; Microsoft Corporation, Redmond, Washington, USA).

### Data Items

The primary outcome of interest was the recovery of knee flexor strength presented as the LSI. We used the recommended cut-off value of ≥ 90% in the LSI to be considered as “recovered” [4]. The second outcome was the possible association between knee flexor strength and second ACL injuries after ACL reconstruction with an HT autograft. The third outcome of interest was the methodology used to measure knee flexor strength, including test apparatus, type of muscle contraction, range of motion, angular velocity, the number of repetitions, rest between attempts, and when the testing was performed in relation to ACL reconstruction.

### Risk of Bias Assessment

Two authors (JH and RP) independently graded the risk of bias of all included studies. To grade the risk of bias for non-randomized studies, the Risk of Bias Assessment Tool for Non-Randomized Studies (RoBANS) was used [17]. Six domains constitute the RoBANS assessment tool: (1) patient selection, (2) confounding variables, (3) measurement of exposure, (4) blinding of the outcome assessments, (5) incomplete outcome data, and (6) selective outcome reporting. Each domain is evaluated and rated as either low risk of bias, unclear risk of bias, or high risk of bias [17]. In the event of disagreement between the two authors (JH and RP), a consensus discussion was held with the senior author (EHS).

To grade the risk of bias for RCTs, the revised version of the Cochrane Risk of Bias tool (RoB (2) was used [18]. The RoB 2 consists of the following domains: (1) risk of bias arising from the randomization process, (2) risk of bias due to deviations from the intended intervention, (3) missing outcome data, (4) risk of bias in the measurement of the outcome, and (5) risk of bias in the selection of the reported result. Each domain includes signal questions with the following responses: “yes,” “probably yes,” “probably no,” “no,” and “no information,” which is summarized to produce a collected risk of bias within each domain. In the end, a summary of the risk of bias for each domain leads to an overall risk of bias for the respective study. Risk of bias was interpreted as follows: (1) low risk of bias if all the domains were judged as low risk of bias, (2) some concerns if no domain was judged as high risk and at least one domain was judged as some concerns, and (3) high risk of bias if at least one domain was judged as high risk of bias or if the study was judged to have some concerns in multiple domains that substantially lowered confidence in the result [19].

### Data Synthesis

To perform the meta-analysis, the LSI (mean ± standard error) from studies that had reported data on knee flexor strength at the angular velocities of 60°/seconds (s) and 180°/s in preoperative tests and at 3 months, 6 months, 12 months, and 24 months postoperatively were pooled as weighted means with standard errors in forest plots separated by the angular velocity of 60°/s and 180°/s created in MedCalc (MedCalc Software Ltd, Ostend, Belgium). The standard error was calculated from the standard deviation and sample size using Microsoft Excel. A confidence interval of 95% was used. The angular velocities of 60°/s and 180°/s, and the preoperative, and 3-month, 6-month, 12-month, and 24-month postoperative timepoints were chosen, as they were most frequently used and consequently yielded more data for pooling. Other velocities and/or other timepoints for assessment were summarized in tables and presented as the LSI (mean + standard deviations).

Assessments ± 1 month of individual follow-ups (3 months, 6 months, 12 months, and 24 months) were included. If studies presented the follow-up time in days, the number of days was divided by 30 to approximate the time in months. If studies presented the follow-up time in years, the number of years was divided by 12 to approximate the time in months. For the sake of homogeneity, the eccentric contraction strength results were not included in the meta-analysis but summarized in tables. Due to high estimated clinical heterogeneity in study design, population, and outcomes, a random effect model was used in the meta-analysis [20]. When performing the meta-analysis, the statistical heterogeneity was calculated and assessed according to the *I*^2^-index with the following reference values: 0–24.9% = no heterogeneity, 25–49.9% = low heterogeneity, 50–74.9% = moderate heterogeneity, and 75–100% = high heterogeneity [20]. To present the association between knee flexor strength and second ACL injuries, and for the methodology of measuring knee flexor strength, a qualitative synthesis methodology was used due to clinical heterogeneity and limited data [21]. First, study characteristics including authors, publication year, population size and age, type of graft, and time of follow-up were summarized in tables. Second, the results of knee flexor strength and second ACL injury were summarized. Finally, for the third aim, the methodology to assess knee flexor strength was summarized in tables to explore whether a consistent theme in methodology might emerge. In the event of missing data in the included studies, an email was sent to the corresponding author.

### Certainty of Evidence Assessment

The Grading of Recommendations Assessment Development and Evaluation (GRADE) working group methodology was applied for assessing certainty of evidence for the outcomes studied [22]. The assessment of certainty of evidence started with evaluating the study designs of included studies for the outcome of interest. Certainty of evidence was defined as “high” for RCTs or non-randomized controlled trials, and “low” for observational studies. In the case of merged study designs for the outcome studied, the certainty of evidence was rated as “low.” Following the assessment of study designs, the certainty of evidence could be downgraded (one or two levels, e.g., from high to moderate or high to low) on the basis of:

(A) Risk of bias assessed by the RoB 2 or RoBANS [17, 19].
(B) Inconsistency assessed by the heterogeneity measured by the *I*^2^-index.
(C) Indirectness by evaluating the generalizability of our results by considering differences in study populations, knee flexor strength assessment methodology, and outcome measures.
(D) Imprecision assessed by the 95% confidence interval range for the pooled outcome and the sample size of included studies for the respective outcome.
(E) Risk of publication bias assessed by a funnel plot.

Finally, on the basis of the assessment above, the certainty of evidence was rated as high, moderate, low, or very low.

## Results

**Fig. 1:**
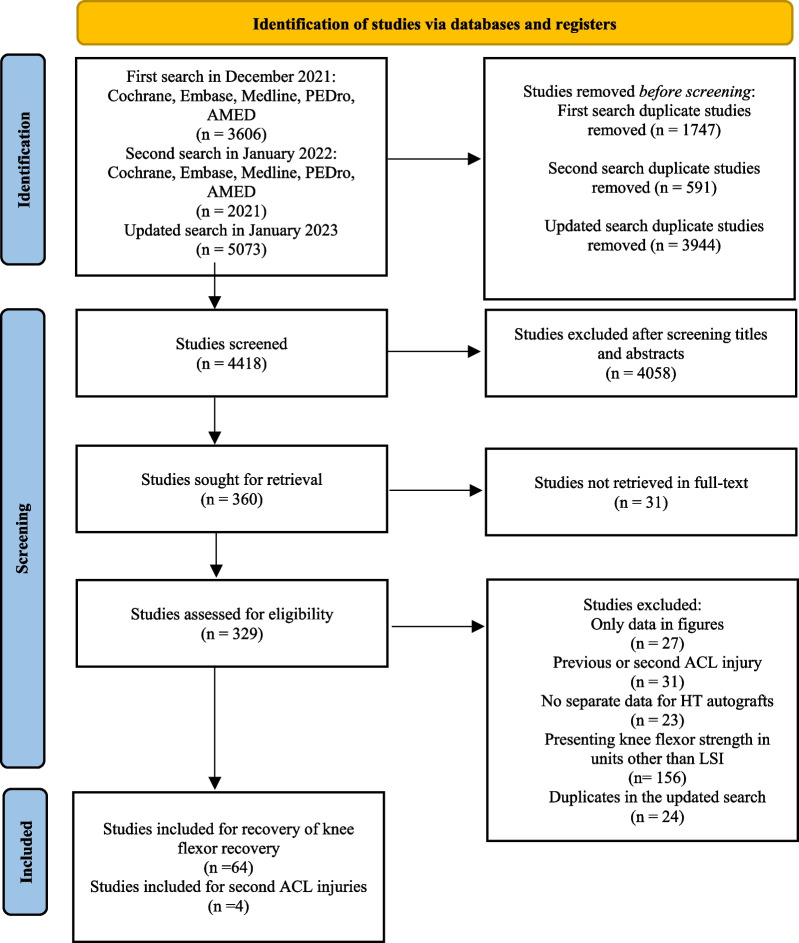
Flowchart of inclusion and exclusion of articles for review. ACL, anterior cruciate ligament; HT, hamstring tendon; LSI, limb symmetry index; *n*, number

The initial search generated 3606 studies and the second 2021 studies, of which 1747 and 591, respectively, were duplicates. The updated search generated 5073 studies, of which 3944 were duplicates. All the studies were uploaded to the Rayyan QCRI web application for a systematic review for the selection process [23]. The initial search was screened by title and abstract and 254 studies were read in full text, of which 53 studies were included to assess the recovery of knee flexor strength symmetry, while 2 studies were included to assess the association between knee flexor strength and subsequent ACL injuries from the two first searches. The updated search resulted in 11 new studies to assess the recovery of knee flexor strength symmetry and 2 studies to assess the association between knee flexor strength and subsequent ACL injuries. Figure 1 illustrates the selection process.

### Recovery of Knee Flexor Strength Symmetry

In total, 8378 patients in the 64 studies were included to assess the recovery of knee flexor strength symmetry. For an overview of study characteristics, see Table 1.

**Table 1:** Study characteristics for studies assessing recovery of knee flexor strength symmetry

Author	Subgroups	Population size, *n*	Men/women	Age, mean ± SD (range)	Autograft	Timepoint of assessments (months)
Araki et al. 2011 [24]	SB	10	5/5	24.7 ± 11.8	STG	12 ± 2.3
	DB	10	5/5	25.2 ± 12.1		13.5 ± 3.2
Baba et al. 2019 [25]	Surgery < 1 months	25	13/12	28.0 ± 11.5	ST	Preop, 24
	Surgery 1–3 months	72	38/34	26.4 ± 11.3		
	Surgery > 3 months	74	48/26	27.5 ± 13.7		
Barenius et al. 2013 [26]	STG	10	8/2	26 ± 9	STG	37 ± 6
	ST	10	8/2	26 ± 7	ST	36 ± 4
Beaudoin et al. 2022 [27]	Ipsilateral graft	20	14/6	41.6 ± 12.4	STG	153.6 ± 20.4
Blucher et al. 2022 [28]	NA	210	100/110	17 (10–19)	STG	12
Carter and Edinger 1999 [29]	ST	33	Missing	Missing	ST	6 (6–7)
	STG	35	Missing	Missing	STG	
Chantrelle et al. 2023 [30]	No anterior knee pain	281	199/82	25.8 ± 6.8	HT	4, 7
Chen et al. 2010 [31]	NA	312	209/103	25 (18–57)	STG	Preop, 55
Chen et al. 2004 [32]	NA	62	39/23	24 (18–52)	STG	Preop, 28
Cristiani et al. 2019 [33]	HT group	3788	Missing	Missing	HT	6
Ebert et al. 2019 [34]	NA	50	32/18	26.3 ± 9.6 (16–49)	STG	12, 24
Ebert et al. 2021 [35]	DB HT graft	69	44/25	30.8 ± 10.6 (16–49)	STG	92.4 ± 7.2 (84–114)
	DB HT graft + LARS	67	45/22	31.1 ± 9.3 (16–49)		94.8 ± 10.8 (84–120)
Ebert et al. 2021 [36]	NA	25	17/8	25.4 ± 8.9	ST	10.8 ± 1.4 (9–12)
Ebert et al. 2022 [37]	Accelerated	22	12/10	24.9 ± 7.1 (16–41)	STG	6, 9, 12, 24
	Control	22	12/10	25.7 ± 7.9 (16–42)		
Fabbriciani et al. 2005 [38]	NA	18	18/0	27.4 (17–40)	STG	6, 24
Fischer et al. 2018 [39]	NA	63	47/16	21.5 ± 6.9 (11–41)	HT	5.4 ± 1.3, 7.5 ± 1.8
Gifstad et al. 2013 [40]	EzLoc	55	36/19	24 (18–45)	STG	24
	Bone Mulch	55	35/20	27 (18–45)		
Guney-Deniz et al. 2020 [41]	HT group	24	18/4	26.7 ± 4.6	HT	13.3 ± 1.8
Hamada et al. 2001 [42]	Single-socket	57	26/31	25 ± 8.5	HT	26.7 ± 3.5
	Bi-socket	49	23/26	24 ± 8.2		
Hanada et al. 2019 [43]	HT group	32	17/15	31 (14–60)	STG	Preop, 6, 12
Harilainen et al. 2005 [44]	Transfix	31	19/12	27 (15–56)	STG	12, 24
	Interference screw	31	23/8	32 (18–49)	ST	
Harilainen et al. 2006 [45]	HT group	48	Missing	Missing	STG	60 (47–79)
Harput et al. 2018 [46]	NA	72	72/0	28.0 ± 7.6	STG	6
Hasebe et al. 2005 [47]	NA	15	8/7	22 ± 4.1	STG	Preop, 27.5 ± 2.5
Holm et al. 2010 [48]	HT group	29	15/14	27 ± 9	STG	120
Högberg et al. 2022 [49]	NA	127	70/57	24.9 ± 8.1	HT	2.5, 4, 8, 12
Inagaki et al. 2013 [50]	ST	61	35/26	28.2 ± 11.9	ST	24
	STG	59	33/26	26.2 ± 10.3	STG	
Iriuchishima et al. 2010 [51]	Standard	14	9/5	28.2 ± 7.6	HT	3, 6, 9
	Accelerated	20	12/8	23.2 ± 4.5		
Johnston et al. 2022 [52]	HT group	70	54/16	20.0 ± 9.0	STG	6, 12
Karlson et al. 1994 [53]	OTT	32	19/13	26.9 ± 7.2	STG	39.6 ± 8.4
	TCC	32	23/9	28.0 ± 9.0		28.8 ± 3.6
Kılınç et al. 2015 [54]	NA	55	54/1	28.18 ± 6.21 (17–40)	HT	23.09 ± 9.08 (9–42)
Koga et al. 2015 [55]	0°	25	18/7	24.6 (14–52)	ST	24
	20°	26	14/12	26.2 (13–63)		
	45°	24	14/10	22.4 (14–42)		
Koga et al. 2015 [56]	SB	25	7/18	24 (14–44)	ST	71 (36–140)
	DB	28	16/12	25 (14–49)		68 (36–136)
Kondo et al. 2012 [57]	ECL	23	12/11	24 (15–45)	STG	24
	ECL-BTB	23	13/10	25 (15–45)		
Kondo et al. 2016 [58]	40° tension	44	30/14	27 (14–57)	HT	29 (24–72)
	30° tension	53	31/22	26 (15–50)		
Kouloumentas et al. 2019 [59]	ST	45	28/17	27.6 ± 11.4	ST	24
	STG	45	27/18	29.7 ± 11.0	STG	
Koutras et al. 2013 [60]	Anteromedial portal	15	15/0	21.5 ± 4	HT	3, 6
	Transtibial portal	36	26/0	24.9 ± 6		
Królikowska et al. 2019 [61]	Supervised < 6 months	77	77/0	29.3 ± 7.4	STG	6.8 ± 1.5
	Supervised > 6 months	66	66/0	29.4 ± 9.0		
Królikowska et al. 2018 [62]	Supervised > 6 months	18	18/0	25.1 ± 5.6	STG	6.9 ± 1.5, 7.8 ± 2.1
Lautamies et al. 2008 [63]	HT group	113	65/48	29 (13–56)	STG	60
Lee et al. 2015 [64]	NA	20	15/5	30.5 (17–51)	STG	Preop, 6, 12
Lee et al. 2016 [65]	HT group	48	44/4	29.9 (17–58)	STG	12, 24
Lesevic et al. 2020 [66]	HT group	66	34/32	Missing	HT	6
Maeda et al. 1996 [67]	NA	41	22/19	24 (16–41)	ST	27 (24–42)
Matsumoto et al. 2006 [68]	HT group	35	15/20	24.4 ± 9.7	STG	80.7 ± 13.2
Murray et al. 2019 [69]	NA	10	2/8	24.6 ± 5.5	STG	6, 12, 24
Nakamura et al. 2002 [70]	ST	49	28/21	24.3	ST	24
	STG	25	6/19	25.7	STG	
Nishio et al. 2018 [71]	> 40 years old	40	17/43	48 (40–71)	ST	24
	< 40 years old	56	31/25	38 (20–39)		
Ogborn et al. 2021 [72]	NA	31	18/13	44.2 ± 10.7	ST	168 ± 52.8
Riesterer et al. 2020 [73]	NA	80	54/26	29.9 ± 9	ST	Preop, 6
Roman et al. 2021 [74]	Adolescents (15–17 years)	89	42/47	16.3 ± 0.8	HT	7.6 ± 1.5
Sanada et al. 2021 [75]	HT group	29	29/0	16.5 (12–20)	HT	5, 8, 12
San Jose et al. 2022 [76]	Early rehabilitation	89	51/38	21 (18–25)	HT	4 (4–5)
	Late rehabilitation	42	Missing	20 (18–25)		10 (9–11)
Sengoku et al. 2022 [77]	ST	41	21/20	21.7 ± 9.2	ST	3, 6
	STG	41	21/20	19.6 ± 7.0	STG	
Severyns et al. 2022 [78]	No graft failure	93	62/31	26.5 ± 9	ST	6
	Graft failure	11	7/4	22.7 ± 6.1		
Sinding et al. 2020 [79]	HT group	43	23/20	28.3 ± 6.2	STG	12
Suh et al. 2021 [80]	Dominant leg	58	40/18	29.5 ± 9.1	HT	Preop, 6, 12
	Non-dominant leg	42	32/10	28.8 ± 9.1		
Tajima et al. 2021 [81]	HT group	43	40/3	24.8 ± 7.3	STG	12
Taketomi et al. 2018 [82]	HT group	23	6/17	32 (15–55)	HT	12
Tanaka et al. 2010 [83]	NA	64	64/0	16.2 (12–29)	ST	Preop, 6
Tim-Yun Ong et al. 2022 [84]	NA	16	8/8	25.7 ± 6	HT	6–9
Tsuda et al. 2009 [85]	Women	50	0/50	23 ± 9 (13–47)	STG	Preop, 3, 6, 12, 18, 37
	Men	32	32/0	26 ± 10 (14–44)		
Ueda et al. 2021 [86]	NA	97	51/46	21.7 ± 8.5	HT	12.2 ± 1 (11–16)
Witvrouw et al. 2001 [87]	HT group	32	17/15	24.6 (17–34)	STG	Preop, 6, 12

DB, double-bundle; ECL, Endobutton-CL; ECL-BTB, Endobutton-CL-BTB; HT, hamstring tendon autograft, unspecified; LARS, Ligament Augmentation and Reconstruction System; NA, not applicable; OTT, over-the-top graft placement; Preop, preoperative; SB, single-bundle; ST, semitendinosus alone; STG, semitendinosus + gracilis; TCC, through the condyle graft placement

#### Risk of Bias Assessment for Knee Flexor Strength Recovery

Of the 64 studies included, 51 (80%) studies were non-randomized and 13 (20%) were RCTs. Of the non-randomized studies, addressing confounding variables and selective outcome reporting were the items with greatest risk of bias (Table 2). Of the 13 RCTs assessed with the Cochrane RoB 2 tool, one study was regarded as having a low risk of bias [59], seven as having some concerns [27, 37, 40, 44, 45, 55, 79], and five as having a high risk of bias [24, 29, 48, 56, 68]. The selection of the reported result was the item with greatest risk of bias (Table 3).

**Table 2:**
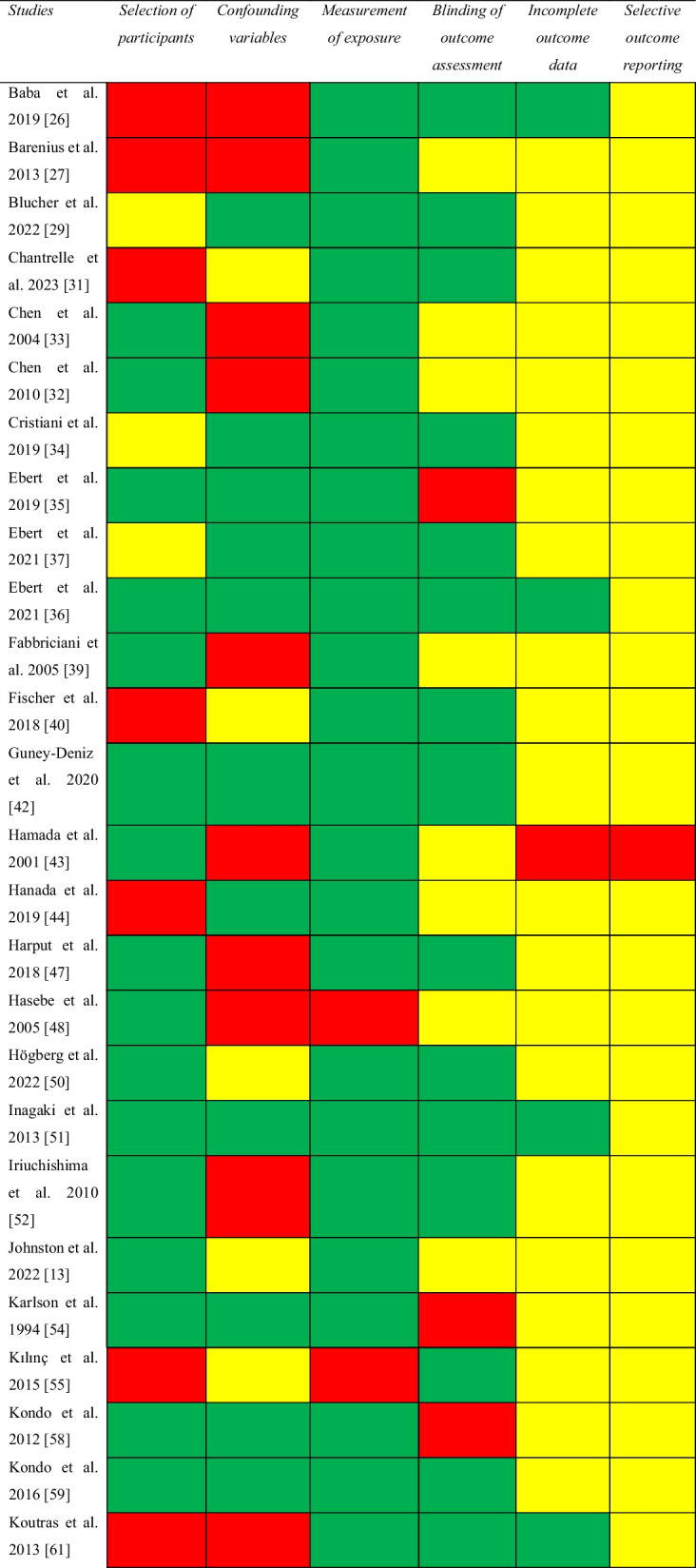

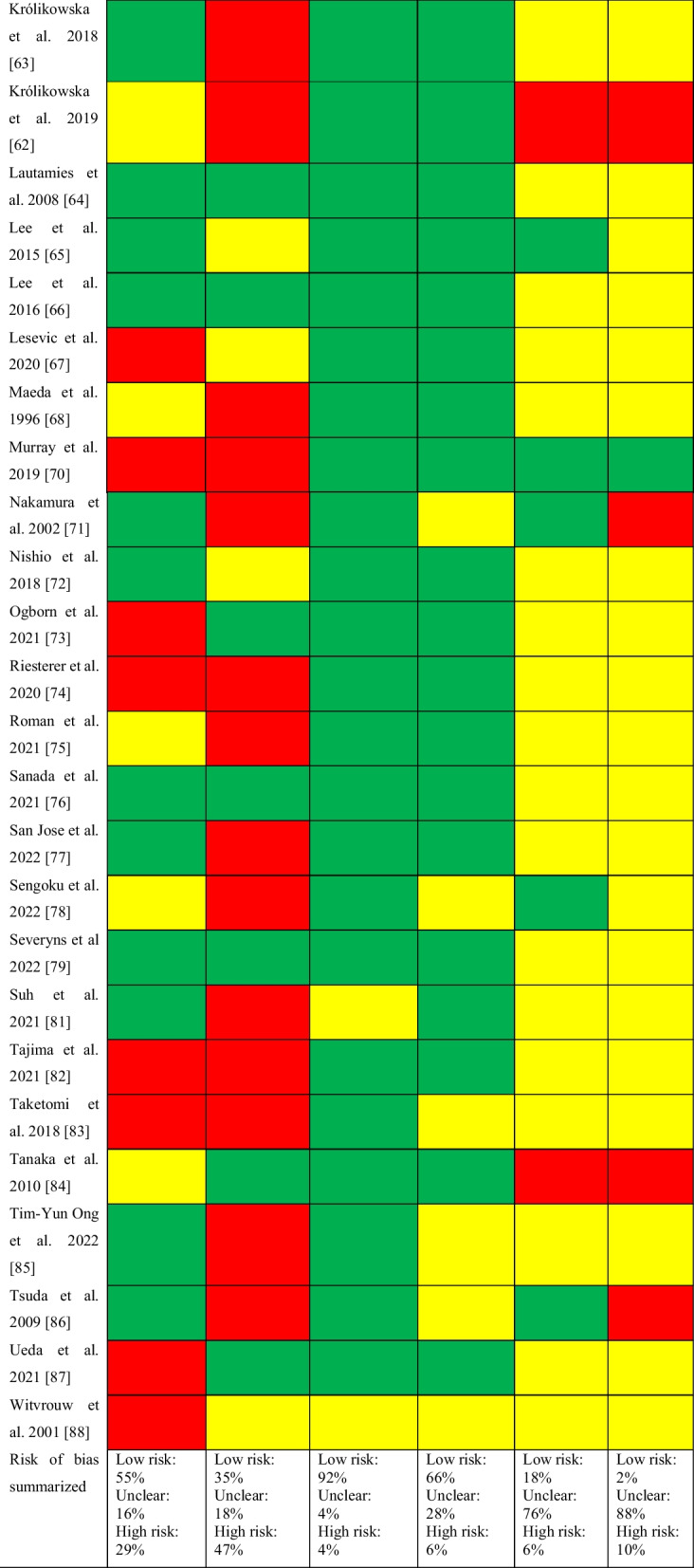

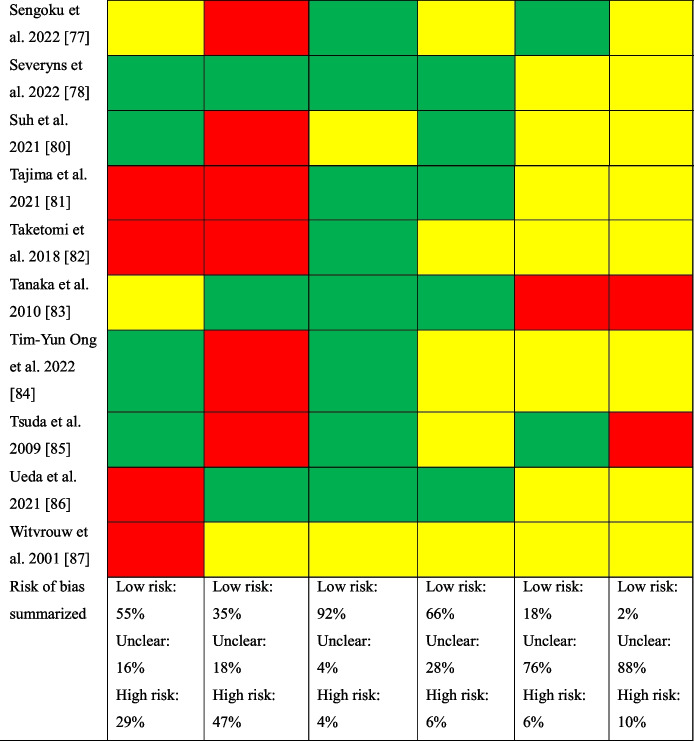
Risk of bias assessed with the risk of bias assessment tool for non-randomized studies (RoBANS)

Green, low risk; yellow, unclear; red, high risk

**Table 3:**
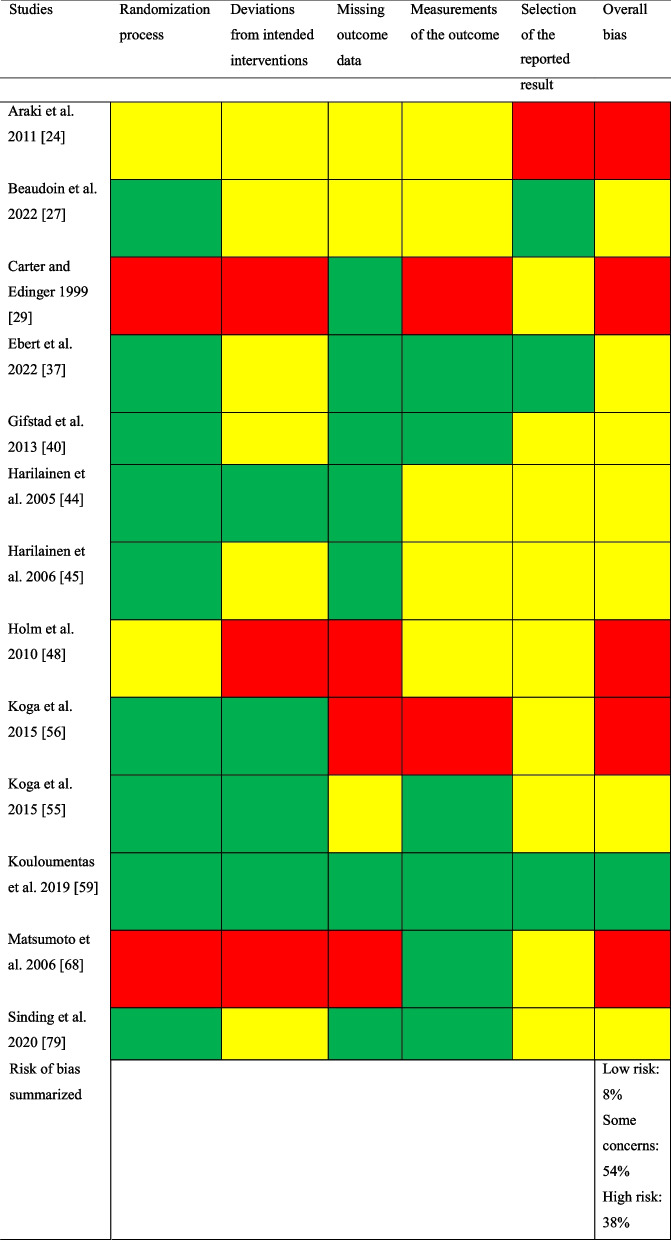
Risk of bias assessed with the Cochrane Risk of Bias tool for randomized controlled trials (ROB 2)

Green, low risk of bias; yellow, some concerns; red, high risk of bias

#### Recovery of LSI for Knee Flexor Strength

There were 36 studies that assessed knee flexor strength symmetry isokinetically with an angular velocity of 60°/s [24, 25, 28, 30, 40–44, 47, 50–52, 55, 57, 59, 60, 62, 64, 65, 69–71, 73–76, 78, 79, 81–83, 85–88] and 21 studies with an angular velocity of 180°/s [28–30, 38, 41, 42, 44, 52, 59–62, 65, 70, 75, 76, 78–80, 88]. For 60°/s, the LSI calculated as the weighted mean with standard error was 80.8% ± 2.5% (95% CI 75.9; 85.7%, *I*^2^ = 89.5%) preoperatively, 81.7% ± 2.8% (95% CI 76.2; 87.2%, *I*^2^ = 95.4%) at 3 months, 88.6% ± 2.0% (95% CI 84.6; 92.6%, *I*^2^ = 93.3%) at 6 months, 89.0% ± 0.9% (95% CI 87.3; 90.7%, *I*^2^ = 66.2%) at 12 months, and 91.7% ± 0.5% (95% CI 90.8; 92.6%, *I*^2^ = 5.4%) at 24 months. The certainty of evidence assessed with the GRADE was very low for all timepoints of assessment except for the 24-month follow-up, of which the certainty of evidence was regarded as low. The inclusion of different study designs, risk of bias, and moderate-to-high statistical heterogeneity lowered the certainty of evidence.

**Fig. 2:**
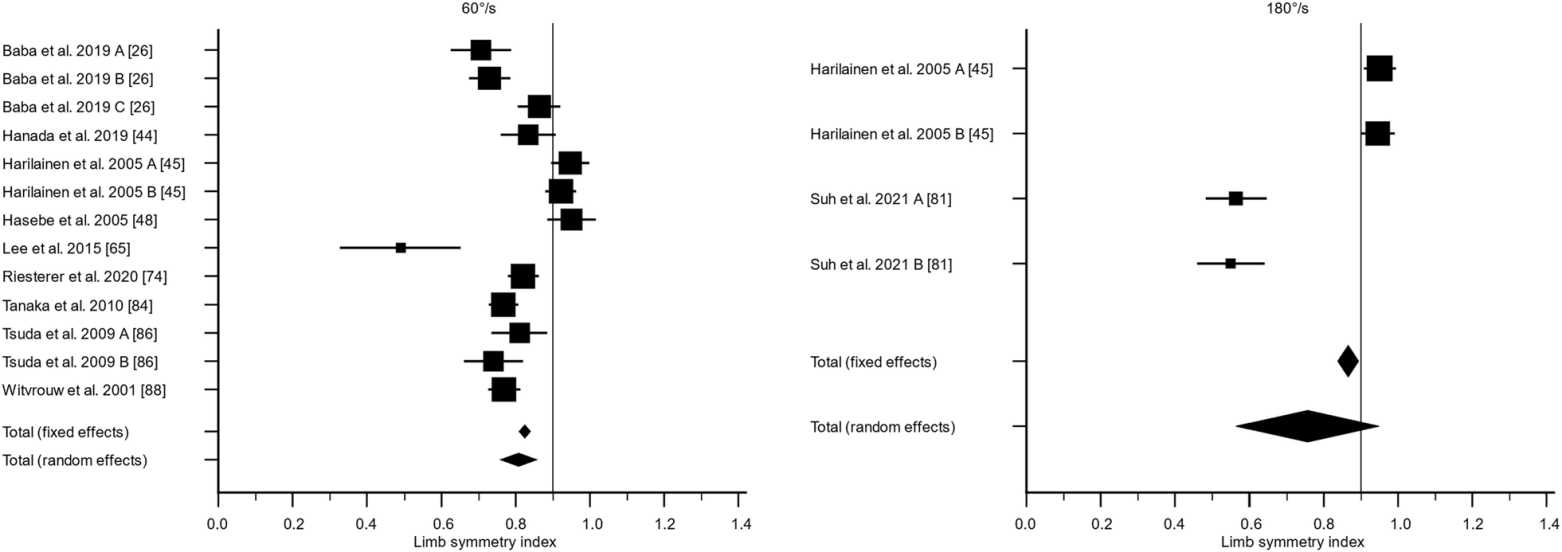
The recovery of knee flexor strength symmetry presented as the limb symmetry index (LSI) preoperatively. The LSI mean value is presented with the standard error and a 95% confidence interval using a random effect model. A limb symmetry index of 90% is represented by the black line. Knee flexor strength at 60°/second was 80.8% ± 2.5% (95% CI 75.9; 85.7%, *I*^2^ = 89.5%) and at 180°/second 75.6% ± 9.8% (95% CI 56.4; 94.8%, *I*^2^ = 97.6%). Baba et al. 2019 A [25] = Surgery < 1 months; Baba et al. 2019 B [25] = Surgery 1–3 months; Baba et al. 2019 C [25] = Surgery > 3 months; Harilainen et al. 2005 A [44] = Transfix; Harilainen et al. 2005 B [44] = Interference screw; Suh et al. 2021 A [80] = Dominant leg; Suh et al. 2021 B [80] = Non-dominant leg; Tsuda et al. 2009 A [85] = Women; Tsuda et al. 2009 B [85] = Men

**Fig. 3:**
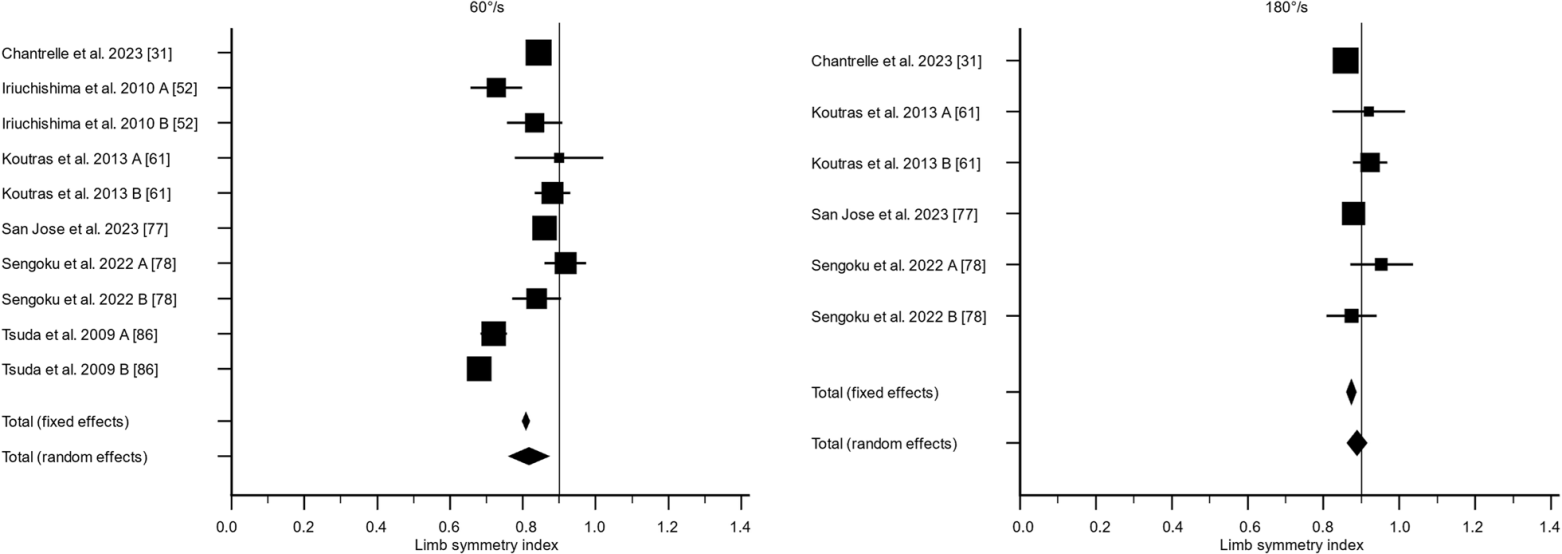
The recovery of knee flexor strength symmetry presented as the limb symmetry index (LSI) at 3 months. The LSI mean value is presented with the standard error and a 95% confidence interval using a random effect model. A limb symmetry index of 90% is represented by the black line. Knee flexor strength at 60°/second was 81.7% ± 2.8% (95% CI 76.2; 87.2%, *I*^2^ = 95.4%) and at 180°/second 88.8% ± 1.4% (95% CI 86.2; 91.5%, *I*^2^ = 58.4%). Iriuchishima et al. 2010 A [51] = Standard rehabilitation; Iriuchishima et al. 2010 B [51] = Accelerated rehabilitation; Koutras et al. 2013 A [60] = Anteromedial portal; Koutras et al. 2013 B [60] = Transtibial portal; Sengoku et al. 2022 A [77] = Semitendinosus alone; Sengoku et al. 2022 B [77] = Semitendinosus + gracilis; Tsuda et al. 2009 A [85] = Women; Tsuda et al. 2009 B [85] = Men

**Fig. 4:**
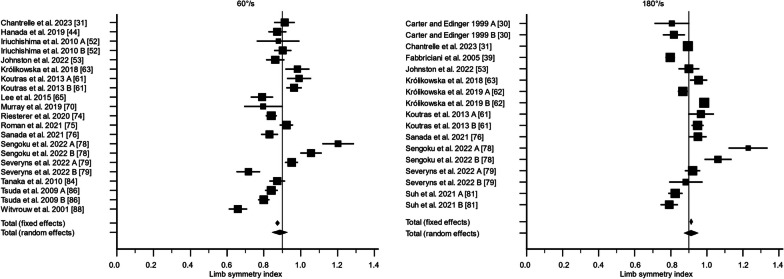
The recovery of knee flexor strength symmetry presented as the limb symmetry index (LSI) at 6 months. The LSI mean value is presented with the standard error and a 95% confidence interval using a random effect model. A limb symmetry index of 90% is represented by the black line. Knee flexor strength at 60°/second was 89.0% ± 0.9% (95% CI 87.3; 90.7%, *I*^2^ = 66.2%) and at 180°/second 91.3% ± 1.9% (95% CI 87.5; 95.1%, *I*^2^ = 94.7%). Carter and Edinger 1999 A [29] = Semitendinosus alone; Carter and Edinger 1999 B [29] = Semitendinosus + gracilis; Iriuchishima et al. 2010 A [51] = Standard rehabilitation; Iriuchishima et al. 2010 B [51] = Accelerated rehabilitation; Królikowska et al. 2019 A [62] = Supervised < 6 months; Królikowska et al. 2019 B [62] = Supervised > 6 months; Koutras et al. 2013 A [60] = Anteromedial portal; Koutras et al. 2013 B [60] = Transtibial portal; Sengoku et al. 2022 A [77] = Semitendinosus alone; Sengoku et al. 2022 B [77] = Semitendinosus + gracilis; Severyns et al. 2022 A [78] = No graft failure; Severyns et al. 2022 B [78] = Graft failure; Suh et al. 2021 A [80] = Dominant leg; Suh et al. 2021 B [80] = Non-dominant leg; Tsuda et al. 2009 A [85] = Women; Tsuda et al. 2009 B [85] = Men

**Fig. 5:**
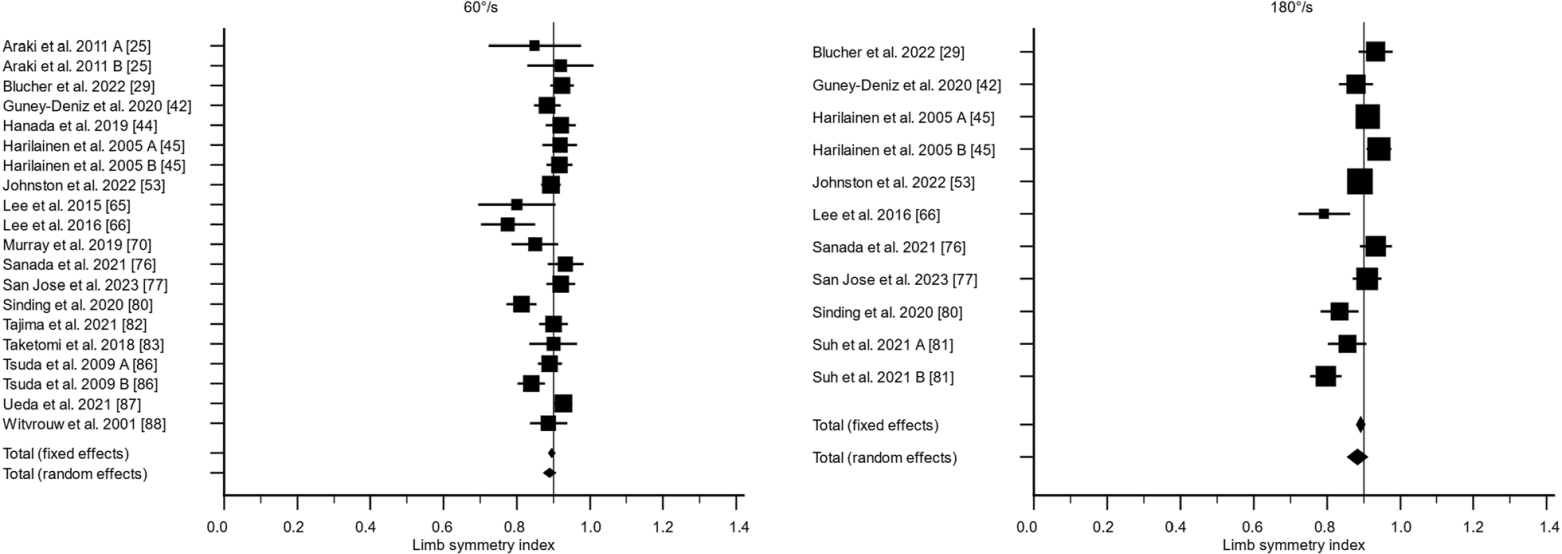
The recovery of knee flexor strength symmetry presented as the limb symmetry index (LSI) at 12 months. The LSI mean value is presented with the standard error and a 95% confidence interval using a random effect model. A limb symmetry index of 90% is represented by the black line. Knee flexor strength at 60°/second was 89.0% ± 0.9% (95% CI 87.3; 90.7%, *I*^2^ = 66.2%) and at 180°/second 88.3% ± 1.4% (95% CI 85.5; 91.1%, *I*^2^ = 80.3%). Araki et al. 2011 A = Single-bundle; Araki et al. 2011 B; Double-bundle; Harilainen et al. 2005 A [44] = Transfix; Harilainen et al. 2005 B [44] = Interference crew; Suh et al. 2021 A [80] = Dominant leg; Suh et al. 2021 B [80] = Non-dominant leg; Tsuda et al. 2009 A [85] = Women; Tsuda et al. 2009 B [85] = Men

**Fig. 6:**
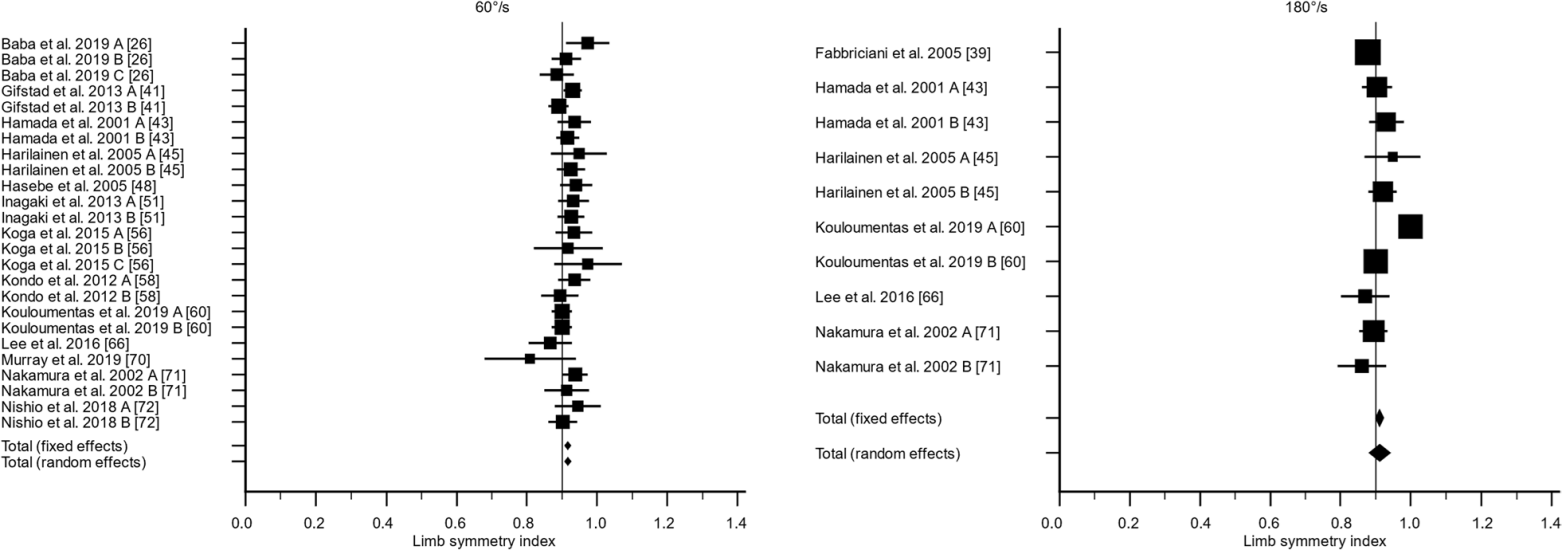
The recovery of knee flexor strength symmetry presented as the limb symmetry index (LSI) at 24 months. The LSI mean value is presented with the standard error and a 95% confidence interval using a random effect model. A limb symmetry index of 90% is represented by the black line. Knee flexor strength at 60°/second was 91.7% ± 0.5% (95% CI 90.8; 92.6%, *I*^2^ = 5.4%) and at 180°/second 91.2% ± 1.5% (95% CI 88.1; 94.2%, *I*^2^ = 82.6%). Baba et al. 2019 [25] A = Surgery < 1 month, Baba et al. 2019 B [25] = Surgery 1–3 months; Baba et al. 2019 C [25] = Surgery > 3 months; Gifstad et al. 2013 A [40] = Ezloc; Gifstad et al. 2013 B [40] = Bone mulch; Hamada et al. 2001 A [42] = Single-socket; Hamada et al. 2001 B [42] = Bi-socket; Harilainen et al. 2005 A [44] = Transfix; Harilainen et al. 2005 B [44] = Interference screw; Inagaki et al. 2013 A [50] = Semitendinosus alone; Inagaki et al. 2013 B [50] = Semitendinosus + gracilis; Koga et al. 2015 A [55] = 0°; Koga et al. 2015 B [55] = 20°; Koga et al. 2015 A [55] = 45°; Kondo et al. 2012 A [57] = Endobutton-CL; Kondo et al. 2012 B [57] = Endobuttion-CL-BTB; Kouloumentas et al. 2019 A [59] = Semitendinosus alone; Kouloumentas et al. 2019 B [59] = Semitendinosus + gracilis; Nakamura et al. 2002 A [70] = Semitendinosus alone; Nakamura et al. 2002 B [70] = Semitendinosus + gracilis; Nishio et al. 2018 A [71] =  > 40 years; Nishio et al. 2018 B [71] =  < 40 years

For 180°/s, the LSI calculated as the weighted mean with standard error was 75.6% ± 9.8% (95% CI 56.4; 94.8%, *I*^2^ = 97.6%) preoperatively, 88.8% ± 1.4% (95% CI 86.2; 91.5%, *I*^2^ = 58.4%) at 3 months, 91.3% ± 1.9% (95% CI 87.5; 95.1%, *I*^2^ = 94.7%) at 6 months, 88.3% ± 1.4% (95% CI 85.5; 91.1%, *I*^2^ = 80.3%) at 12 months, and 91.2% ± 1.5% (95% CI 88.1; 94.2%, *I*^2^ = 82.6%) at 24 months (Figs. 2, 3, 4, 5, 6). The certainty of evidence assessed with the GRADE was very low for all timepoints of assessment. The inclusion of different study designs, risk of bias, and moderate-to-high statistical heterogeneity lowered the certainty of evidence.

Furthermore, 14 studies presented data for knee flexor strength symmetry at 90°/s, 240°/s, or 300°/s instead of 60°/s or 180°/s [27, 29, 33–37, 46, 48, 49, 54, 66, 72, 87], while 2 studies used three velocities (60°/s, 90°/s, and 180°/s) and did not explicitly report whether the results, as a mean LSI, were an average based on all three velocities or the result of a specific velocity [31, 32]. A total of 18 studies presented data at different follow-ups (ranging from 6 to 168 ± 52.8 months) than preoperatively and at 3 months, 6 months, 12 months, and 24 months [26, 27, 35, 39, 45, 48, 51, 53, 56, 58, 62, 63, 67, 68, 72, 75, 84, 85]. Studies presenting data for knee flexor strength symmetry as the LSI at velocities other than 60°/s and 180°/s and at timepoints other than preoperatively and 3 months, 6 months, 12 months, and 24 months postoperatively are presented in Additional file 2: Complementary Result Tables, Table S1.

### Knee Flexor Strength and Second ACL Injuries

In total, there were 610 patients in four studies investigating the association between knee flexor strength and second ACL injuries (Table 4). All the studies compared knee flexor strength in patients who sustained a second ACL injury with those who did not. In addition, Yamanashi et al. [89] performed a covariance structure analysis and presented low negative covariance values of −0.09 for the hamstring-to-quadriceps ratio at 3 months and −0.1 at 6 months. Collectively, there was insufficient and contradictory data to allow firm conclusions to be drawn in terms of the association between knee flexor strength and second ACL injuries.

**Table 4:** Study characteristics for studies assessing association between knee flexor strength and second ACL injuries

Studies	Population size, *n*	Men/ women	Age, mean ± SD (range)	Graft	Follow-up duration after ACL reconstruction (months)
Blucher et al. 2022 [28]	210	100/110	17 (10–19)	STG	> 24
Severyns et al. 2022 [78]	104	68/35	22.7 ± 6.1 (re-tears) 26.5 ± 9.0 (no re-tears)	ST	42.3 ± 12.1
Tanaka et al. 2010 [83]	64	0/64	16.2 (12–29)	ST	24
Yamanashi et al. 2019 [89]	232	101/131	26.1 ± 11.9	HT	18.9 ± 9.7

ACL, anterior cruciate ligament; HT, hamstring tendon autograft, unspecified; *N*, numbers; NA, not applicable; SD, standard deviation; ST, semitendinosus only used; STG, semitendinosus + gracilis

#### Risk of Bias Assessment for Studies Investigating Knee Flexor Strength and Second ACL Injuries

The studies included in the analysis of the association between knee flexor strength and second ACL injuries consisted of one case series [83], two retrospective cohort studies [78, 89], and one case–control study [28]. Incomplete outcome data and selective outcome reporting were the items with greatest risk of bias (Table 5).

**Table 5:**
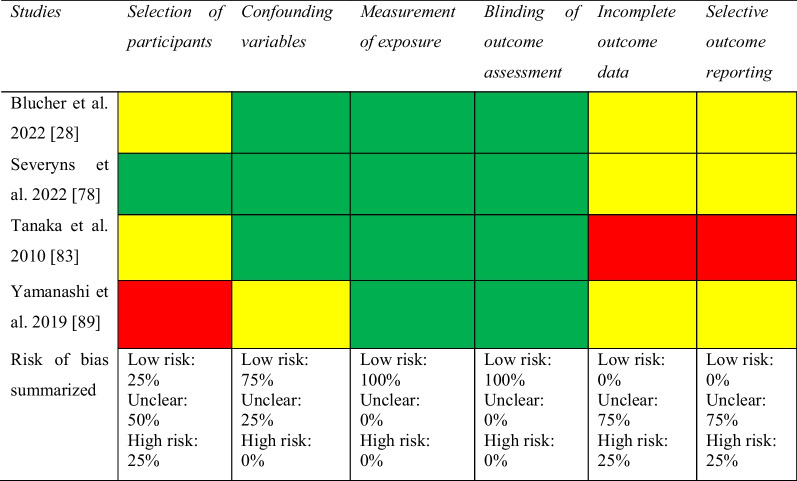
Risk of bias assessed with the Risk of Bias Assessment Tool for Non-Randomized Studies (RoBANS)

Green, low risk of bias; yellow, some concerns; red, high risk of bias

#### Association Between Knee Flexor Strength and Second ACL Injuries

A total of 72 second ACL injuries were reported across the four included studies, of which 63 were ipsilateral and 9 contralateral [28, 78, 83, 89]. The studies presented data for knee flexor strength for the ACL re-injured group ranged from 3 months [89] to 12 months postoperatively [28] (Table 6).

**Table 6:** Knee flexor strength for patients sustaining second ACL injuries

Studies	Ipsilateral/contralateral injury, *n*	Timepoint of assessment (months)	Time at RTS (months)	Time of injury after ACL reconstruction (months)	Group	Knee flexor strength, LSI (%)	Absolute knee flexor strength values (Nm)	HQ-strength ratio, mean ± SD
Blucher et al. 2022 [28]	19/0	12	12 (70%)	23 (13–36)	Second ACL injury, *n* = 19	93.6% ± 9.4% (60°/s) 92.2% ± 16.0% (180°/s)	–	–
					No Second ACL injury, *n* = 191	90.1% ± 19.4% (60°/s) 91.4% ± 16.2% (180°/s)		
Severyns et al. 2022 [78]	11/0	6	6^1^	11.5 (7–19)	Second ACL injury, *n* = 11	**71.6% ± 10.9% (60°/s)** 88.4% ± 15.5% (180°/s)	**66.1 ± 26.2** 61.2 ± 24.2	–
					No second ACL injury, *n* = 93	**95.0% ± 17.5% (60°/s)** 92.1% ± 20.7% (180°/s)	**94.1 ± 32.4** 72.6 ± 28.4	
Tanaka et al. 2010 [83]	6/0	6	9.8	11.7 (8.0–15.7)	Second ACL injury, *n* = 6	90.9% ± 13.7%	63.7 ± 25.5	51.8% ± 8.0%
					No second ACL injury, *n* = 58	87.0% ± 17.5%	55.8 ± 15.7	53.2% ± 15.5%
Yamanashi et al. 2019 A [89] ^2^	27/9	3	6–12^1^	Missing	Second ACL injury, *n* = 14	–	81.7 ± 16.4	**43.0% ± 8.0%**
					No second ACL injury, *n* = 131		77.2 ± 64.2	**48.6% ± 11.6%**
Yamanashi et al. 2019 B [89] ^2^	27/9	6	6–12^1^	Missing	Second ACL injury, *n* = 12	–	81.6 ± 18.9	**40.3% ± 7.0%**
					No second ACL injury, *n* = 115		88.3 ± 22.1	**50.8% ± 11.4%**

Bold numbers indicates significantly lower for reinjury group, *p* < 0.05

^1^ Numbers relate to when the patients were permitted to return to sports

^2^ Knee flexor strength is only presented for those who sustained an ipsilateral injury

ACL, anterior cruciate ligament; HQ, hamstring-to-quadriceps; LSI, limb symmetry index; *n*, numbers; NA, not applicable; Nm, Newton meters; RTS, return to sport; SD, standard deviation. Yamanashi et al. [89] A refers to the 3-month values while B refers to the 6-month values

### Methodology for Measuring Knee Flexor Strength

To assess knee flexor strength, 22 studies used a Cybex dynamometer [25, 29, 31, 32, 42, 47, 48, 51, 54–58, 65, 67, 68, 70, 71, 82, 83, 85, 87], 19 used a Biodex dynamometer [24, 26, 27, 40, 41, 43, 49, 59, 64, 66, 69, 72, 75, 80, 81, 84, 88–90], 9 used a HUMAC dynamometer [28, 30, 52, 61, 62, 73, 74, 76, 79], 4 used an IsoSport dynamometer [34–37], 3 used a Lido Multijoint dynamometer [44, 45, 63], 2 used a Genu Plus dynamometer [38, 86], 2 used a Con-Trex dynamometer [39, 78], 1 used a Kin-Com dynamometer [50], 1 used an Orthrotron [53], and 1 study did not report the test equipment that was used [60]. A total of 23 studies specified the contraction mode, with 21 studies using a concentric contraction [27, 34, 36, 39, 41, 49, 52, 54, 61–64, 66, 72, 73, 76, 80, 82, 84, 85, 89], 2 studies investigating both concentric and eccentric contractions [46, 79], and 42 studies not specifying the contraction mode [24–26, 28–33, 35, 37, 38, 40, 43–45, 47, 50, 51, 53, 55–60, 65, 67–71, 74, 75, 78, 81, 83, 86–88]. Regarding the position at the time of testing, 22 studies specified the test position as a seated position [26–30, 36, 41, 46, 49, 51, 52, 61–64, 72–74, 76, 78, 80, 84], 2 studies used a supine position [27, 72], and 43 studies did not specify the position [24, 25, 31–35, 37–40, 42–45, 47, 48, 50, 53–60, 65–71, 75, 79, 81–83, 85–89].

Collectively, the methods of knee flexor strength assessment appear to vary substantially. Further information regarding the methodology for measuring knee flexor strength is summarized in Additional file 2: Complementary Result Tables, Table S2 for studies assessing the recovery of knee flexor strength, and in Additional file 2: Complementary Result Tables, Table S3 for studies assessing the association between knee flexor strength and second ACL injuries.

## Discussion

The main finding of this systematic review and meta-analysis was, based on very low to low certainty of evidence, an incomplete recovery of knee flexor strength symmetry for up to a year after ACL reconstruction with an HT autograft, with regard to the 90% LSI reference cut-off value to be classified as “recovered.” The isokinetic knee flexor strength LSI then appears to be recovered at 2 years after ACL reconstruction at both 60°/s and 180°/s of angular velocity. Despite knee flexor strength asymmetry persisting as late as 1 year after ACL reconstruction, it is still unknown whether knee flexor strength asymmetry affects the occurrence of a second ACL injury, as there was a general lack of data on the association between knee flexor strength and second ACL injuries. In addition to the lack of reported second ACL injuries, the heterogeneity in the methodology used to measure knee flexor strength after ACL reconstruction with HT autografts presents a problem for scientists and clinicians alike. Given the fairly well-accepted role of the knee flexors as synergists to the ACL, the need to bridge this evidence gap is apparent.

### Knee Flexor Strength Recovery

In our systematic review and meta-analysis, we used the commonly recommended cut-off value of ≥ 90% in LSI for an individual to be classified as “recovered.” At 1 year after ACL reconstruction, the average LSI for concentric knee flexor strength at an angular velocity of 60°/s was 89.0% ± 0.9% (95% CI 87.3; 90.7%), increasing to 91.7% ± 0.5% (95% CI 90.8; 92.6%) at 2 years. However, while the recovery of knee flexor strength at 1 year was regarded as “incomplete” and at 2 years as “complete,” the clinical relevance of the difference between 89.0% and 91.7% is questionable. The knee flexor strength recovery after ACL reconstruction with an HT autograft is not well defined and the logical foundation for the recommendation of a cut-off value of ≥ 90% in LSI is not entirely clear, despite being routinely used. First, one may argue that a higher angular velocity, e.g., ≥ 180°/s, imposes a different demand for knee flexor strength than angular velocities of ≤ 60°/s. Undheim et al. [91] reported that torque output increases when angular velocities decrease and thus, to assess maximum strength, angular velocities of ≤ 60°/s were recommended. On the contrary, the differences between 60°/s and 180°/s for up to 2 years after ACL reconstruction were very small and can hardly be considered clinically relevant. Furthermore, the recommendation of ≥ 90% in the LSI for knee flexor strength after ACL reconstruction does not take into account the persistent graft differences in donor-site morbidity. Harvesting the semitendinosus tendon alone or in combination with the gracilis will likely result in a change in morphological properties upon regeneration, and the tendon often inserts more proximally compared with preoperatively [92]. The altered structure of the semitendinosus muscle and tendon will in turn influence the mechanical properties and consequently influence the assessment of knee flexor strength, e.g., with larger knee flexor deficits at more extended hip angles and deeper knee angles [12]. It has been suggested that the biceps femoris long and short head, as well as the semimembranosus, may compensate for the semitendinosus during knee flexor strength assessments [93]. Hence, it can be argued that the LSI cut-off value for knee flexor strength should be even higher in patients with HT autografts, with the changes in the morphological and mechanical properties of the semitendinosus in mind. Importantly, we found that a persisting knee flexor strength deficit defined as < 90% LSI at 1 year has the potential to recover beyond 1 year after ACL reconstruction with HT autograft. Patients with a persistent knee flexor strength deficit between 12 months and 24 months after ACL reconstruction can increase their strength significantly with progressive strength training [94]. Additionally, patients randomized to an accelerated rehabilitation protocol may recover their knee flexor strength symmetry (≥ 90% LSI) as early as 6 months postoperatively without adverse events [37]. Taken together, these considerations suggest there might be an underloading issue in patients with ACL reconstruction treated with HT autograft resulting in persisting knee flexor strength asymmetry. Consequently, on the basis of our findings and the literature, earlier introduction and with a progressive increase in knee flexor strength demands are advocated to resolve the impairments in knee flexor strength after ACL reconstruction with HT autografts.

### Knee Flexor Strength and Second ACL Injuries

A safe return to sport or a return to knee-strenuous activity and minimizing the risk of a second ACL injury are the ultimate aims of clinicians with each unique patient. Recovering ≥ 90% in the LSI is frequently used as part of the assessment criteria prior to returning to sports or knee-strenuous activities [7]. While it appears logical that recovering knee flexor strength LSI to ≥ 90% would be protective, as the knee flexors act as synergists to the ACL, only 4 of 4418 unique identified studies reported knee flexor strength separately for patients who sustained a second ACL injury [28, 78, 83, 89]. Moreover, although several studies that reported second ACL injuries were identified, these studies unfortunately excluded patients who sustained second ACL injuries from the knee flexor strength assessments during the study process, contributing to the limited available data regarding the possible protective role of knee flexor strength in relation to second ACL injuries [75, 95–98]. Interestingly, in a cohort of 210 patients after ACL reconstruction with an HT autograft, Blucher et al. [28] reported that only 45–47% managed to pass ≥ 90% in LSI for knee flexor strength at 1 year assessed isokinetically at both angular velocities of 60°/s and 180°/s. However, there was no significant difference in LSI for knee flexor strength between patients who sustained a second ACL and those who did not (92–94% versus 90–92%, respectively) [28]. In contrast, Severyns et al. [78] reported a significantly lower LSI for knee flexor strength in patients who would go on to sustain a second ACL injury at 6 months compared with patients who did not (71.6% ± 10.9% versus 95.0% ± 17.5%) in a cohort of 104 patients. Collectively, there is a remarkable lack of data regarding the proposed role of strong knee flexors in reducing the risk of second ACL injuries. It is therefore yet to be determined whether passing the cut-off value of ≥ 90% in the LSI for knee flexor strength is important when it comes to reducing second ACL injuries.

One of the included studies presented a significantly reduced hamstring-to-quadriceps strength ratio at 3 months and 6 months for patients who sustained an ipsilateral ACL injury compared with patients who did not sustain a second ACL injury [89]. A lower hamstring-to-quadriceps strength ratio has previously been reported as a risk factor for a primary ACL injury in females [99] and for a second ACL injury among both patellar tendon and HT autografts [6]. Consequently, the relationship between the knee extensors and the flexors may be of greater importance in reducing the risk of second ACL injuries than striving to achieve ≥ 90% LSI knee flexor strength. However, the way we should measure knee flexor strength and how strong the knee flexors should be in relation to the knee extensors to reduce second ACL injuries are other as yet unanswered questions. In addition, whether the hamstring-to-quadriceps ratio and/or knee flexor strength symmetry are important in reducing the risk of second ACL injuries is currently also unclear, due to the general lack of data.

### Knee Flexor Strength Measurement Methodology

There was both great heterogeneity and a general lack of detail in the methodology for measuring knee flexor strength, with only 34% of the included studies specifying which position was used for assessing knee flexor strength. This is a matter of concern, as hip position will affect the peak torque of the reconstructed limb after ACL reconstruction with an HT autograft, with a more extended hip position leading to greater knee flexor strength deficits compared with a more flexed position [12]. Consequently, the fact that so many studies did not specify the test position induces some degree of uncertainty in the interpretation of knee flexor strength symmetry values in the present systematic review and meta-analysis. In addition, there was a lack of reporting of the type of muscle contraction, range of motion, number of repetitions, and rest between each set when there was more than one set. Worryingly, 8 years ago, Undheim et al. [91] reported that there was no standardized isokinetic protocol for measuring strength after ACL reconstruction, which still appears to be the case.

### Limitations

Firstly, our choice to summarize studies with knee flexor strength assessments with the velocities of 60°/s and 180°/s in a forest plot was made as they were the most frequently used velocities and consequently enabled more studies to be included in the meta-analysis. However, the sample sizes for the included studies in the meta-analysis were small in overall terms, ranging from 10 to 281 individuals. The exclusion of studies with languages other than English, and which used other velocities in the pooling of data involved studies with larger sample sizes than the included studies, e.g., Cristiani et al. [33] with 3788 individuals, thereby missing important information on knee flexor strength recovery. Secondly, the inclusion of different study designs together with the heterogeneity in the methodology for knee flexor strength assessment may limit the internal validity of our results. Only 35% of the included studies specified the contraction type, 34% specified the test position, and 52% specified how many repetitions the patients performed during the knee flexor strength assessment. The lack of reporting of methodology for assessing knee flexor strength not only affects the investigation of knee flexor strength recovery, but also further limits the interpretation of whether knee flexor strength influences second ACL injuries. To address the heterogeneity in methodology, statistical heterogeneity was assessed with the *I*^2^ index ranging from 58.4% to 97.6%, except for 60°/second at 24 months, with a value of 5.4%. Consequently, the overall statistical heterogeneity was high, indicating variability in the available data, which may affect the certainty of our results. Furthermore, there was a major limitation in the number of available studies to address the second study aim relating to the association between knee flexor strength and second ACL injuries. In 3 of 4 studies, the time range for assessing knee flexor strength was 3–6 months after ACL reconstruction, although the typical time frame for a return to sport is 9–12 months [100]. This means that we do not know the level of knee flexor strength at the time of a return to sport or the timepoint of the second ACL injury. Finally, the low to very low certainty of evidence assessed by GRADE warrants further caution in the interpretation of our results.

### Future Research

Future research should firstly summarize the methodology used to reach a consensus on how knee flexor strength should be measured after ACL reconstruction in patients with HT autografts. After reaching a consensus, the second step should be to investigate whether knee flexor strength symmetry is an important factor for knee function and the occurrence of a second ACL injury, or whether the strength relationship between the knee extensors and flexors is a better milestone to strive for.

## Conclusions

The recovery of knee flexor strength symmetry appears to take up to 2 years after ACL reconstruction using HT autograft considering ≥ 90% in LSI with a low to very low certainty of evidence. The relevance of persistent knee flexor asymmetry at 1 year in terms of the risk of second ACL injuries is not known due to limited data. Consequently, standardized assessments and more research are needed to clarify whether achieving knee flexor strength symmetry influences the occurrence of a second ACL injury.

### Supplementary Information

**Additional file 1.** The Search Strategy.

**Additional file 2.** Complementary Result Tables.

## Data Availability

All included studies constituting the present systematic review and meta-analysis are cited in the manuscript. The dataset is available upon reasonable request.

## Abbreviations

ACL: Anterior cruciate ligament
HT: Hamstring tendon
LSI: Limb symmetry index
RoBANS: Risk of bias assessment tool for non-randomized studies
RCT: Randomized controlled trial
ROB: Risk of bias
