# Supplementary material for: Restoring Knee Flexor Strength Symmetry Requires 2 Years After ACL Reconstruction, But Does It Matter for Second ACL Injuries? A Systematic Review and Meta-analysis
Source: Sports Med Open. 2024 Jan 5;10:2. doi: 10.1186/s40798-023-00666-5 (PMC10769975; doi:10.1186/s40798-023-00666-5)
Supplement: Supplementary file 1 — Additional file 1. The Search Strategy. [file 40798_2023_666_MOESM1_ESM.docx]

**Online resources**

**Online resource 1**

First search in December 2021.

Antal unika träffar

(n = 1859)

Dubbletter
(n = 1747)

Totalt
(n = 3606)

Cochrane Library

(n = 2 review

n = 499 trials)

Pedro

(n = 9)

Embase

(n = 1594)

Medline

(n = 1400)

AMED

(n = 102)

**Database:** MEDLINE (OVID)

**Date:** 2021-12-02

**No of results:** 1400 ref

| **#** | **Searches** | **Results** |
| --- | --- | --- |
| 1 | exp Anterior Cruciate Ligament/ | 11,524 |
| 2 | ACL.ab,kw,ti. | 18,291 |
| 3 | (anterior adj5 cruciate adj5 ligament*).ab,kw,ti. | 21,030 |
| 4 | 1 or 2 or 3 | 27,954 |
| 5 | (surgical* or surgery or surgeries or reconstruction* or reconstructive or reconstructed or  repair).ab,kw,ti. | 2,424,589 |
| 6 | 4 and 5 | 17,317 |
| 7 | exp Anterior Cruciate Ligament Reconstruction/ | 6,216 |
| 8 | 6 or 7 | 17,725 |
| 9 | Hamstring Tendons/tr | 374 |
| 10 | Hamstring Muscles/tr | 65 |
| 11 | 9 or 10 | 434 |
| 12 | (Hamstring or semitendinosus).ab,kw,ti. | 10,251 |
| 13 | (graft or autograft or transplant* or tissue).ab,kw,ti. | 2,074,827 |
| 14 | 12 and 13 | 3,608 |
| 15 | 11 or 14 | 3,694 |
| 16 | exp treatment outcome/ | 1,160,264 |
| 17 | exp "Recovery of Function"/ | 57,273 |
| 18 | exp "Range of Motion, Articular"/ | 56,683 |
| 19 | ("range of motion" or recover* or outcome or function* or flexion or extension).ab,kw,ti. | 5,618,085 |
| 20 | 16 or 17 or 18 or 19 | 6,384,571 |
| 21 | (Muscle or flexor).ab,kw,ti. | 693,757 |
| 22 | strength.ab,kw,ti. | 325,889 |
| 23 | 21 and 22 | 47,448 |
| 24 | 20 or 23 | 6,402,851 |
| 25 | 8 and 15 and 24 | 1,573 |
| 26 | (Letter or Comment or Editorial or Case Reports).pt. | 4,023,224 |
| 27 | 25 not 26 | 1,507 |
| 28 | (animals not (animals and humans)).sh. | 4,890,387 |
| 29 | 27 not 28 | 1,480 |
| 30 | limit 29 to (danish or english or norwegian or swedish) | 1,400 |

**exp/** = term from the Medline controlled vocabulary, including terms found below this term in the MeSH hierarchy

**/** = term from the Medline controlled vocabulary, does not include terms found below this term in the MeSH hierarchy

**.ti,ab,kf**. = title, abstract and author keywords

**adjx** = within x words, regardless of order

***** = truncation of word for alternate endings

**Database:** EMBASE (OVID)

**Date:** 2021-12-03

**No of results:** 1400 ref

| **#** | **Search** | **Results** |
| --- | --- | --- |
| 1 | exp Anterior Cruciate Ligament/ | 12,658 |
| 2 | ACL.ab,kw,ti. | 23,963 |
| 3 | (anterior adj5 cruciate adj5 ligament*).ab,kw,ti. | 24,780 |
| 4 | 1 or 2 or 3 | 35,621 |
| 5 | (surgical* or surgery or surgeries or reconstruction* or reconstructive or reconstructed or repair).ab,kw,ti. | 3,192,584 |
| 6 | 4 and 5 | 21,399 |
| 7 | exp Anterior Cruciate Ligament Reconstruction/ | 12,609 |
| 8 | 6 or 7 | 22,923 |
| 9 | *hamstring tendon/ | 308 |
| 10 | *hamstring muscle/ | 764 |
| 11 | 9 or 10 | 1,066 |
| 12 | (Hamstring or semitendinosus).ab,kw,ti. | 12,065 |
| 13 | (graft or autograft or transplant* or tissue).ab,kw,ti. | 2,761,633 |
| 14 | 12 and 13 | 4,389 |
| 15 | 11 or 14 | 5,061 |
| 16 | exp treatment outcome/ | 1,879,669 |
| 17 | exp "Recovery of Function"/ | 52,815 |
| 18 | exp "Range of Motion, Articular"/ | 99,210 |
| 19 | ("range of motion" or recover* or outcome or function* or flexion or extension).ab,kw,ti. | 7,165,355 |
| 20 | 16 or 17 or 18 or 19 | 8,316,698 |
| 21 | (Muscle or flexor).ab,kw,ti. | 859,090 |
| 22 | strength.ab,kw,ti. | 377,207 |
| 23 | 21 and 22 | 65,993 |
| 24 | 20 or 23 | 8,340,819 |
| 25 | 8 and 15 and 24 | 2,069 |
| 26 | limit 25 to (books or conference abstract or "conference review" or editorial or erratum or letter or short survey or tombstone) | 324 |
| 27 | 25 not 26 | 1,745 |
| 28 | (animal not (animal and human)).sh. | 1,125,235 |
| 29 | 27 not 28 | 1,733 |
| 30 | limit 29 to (danish or english or norwegian or swedish) | 1,593 |

**exp/** = term from the Medline controlled vocabulary, including terms found below this term in the MeSH hierarchy

**/** = term from the Medline controlled vocabulary, does not include terms found below this term in the MeSH hierarchy

**.ti,ab,kf**. = title, abstract and author keywords

**adjx** = within x words, regardless of order

***** = truncation of word for alternate endings

**Database:** The Cochrane Library
**Date:** 2021-12-10
**No of results:** 501 ref

*Cochrane reviews: 2
Cochrane protocols: 0
Trials: 499
Editorials: 0
Special collections: 0
Clinical answers: 0*

| **ID** | **Search** | **Hits** |
| --- | --- | --- |
| #1 | MeSH descriptor: [Anterior Cruciate Ligament] explode all trees | 670 |
| #2 | (ACL):ti,ab,kw | 2,154 |
| #3 | ("anterior cruciate ligament"):ti,ab,kw | 2,846 |
| #4 | #1 OR #2 OR #3 | 3,292 |
| #5 | (surgical* or surgery or surgeries or reconstruction* or reconstructive or reconstructed or repair):ti,ab,kw | 273,197 |
| #6 | #4 AND #5 | 2,696 |
| #7 | MeSH descriptor: [Anterior Cruciate Ligament Reconstruction] explode all trees | 471 |
| #8 | #6 OR #7 | 2,697 |
| #9 | MeSH descriptor: [Hamstring Tendons] explode all trees and with  qualifier(s): [transplantation – TR] | 38 |
| #10 | MeSH descriptor: [Hamstring Muscles] explode all trees and with qualifier(s): [transplantation - TR] | 8 |
| #11 | #9 OR #10 | 44 |
| #12 | (Hamstring or semitendinosus):ti,ab,kw | 2,420 |
| #13 | (graft or autograft or transplant* or tissue):ti,ab,kw | 151,359 |
| #14 | #12 AND #13 | 788 |
| #15 | #11 OR #14 | 788 |
| #16 | MeSH descriptor: [Treatment Outcome] explode all trees | 148,566 |
| #17 | MeSH descriptor: [Recovery of Function] explode all trees | 5,583 |
| #18 | MeSH descriptor: [Range of Motion, Articular] explode all trees | 5,185 |
| #19 | ("range of motion" or recover* or outcome or function* or flexion or extension):ti,ab,kw | 756,616 |
| #20 | #16 OR #17 OR #18 OR #19 | 761,170 |
| #21 | (muscle OR flexor):ti,ab,kw | 79,751 |
| #22 | (Strength):ti,ab,kw | 39,858 |
| #23 | #21 AND #22 | 22,020 |
| #24 | #20 OR #23 | 766,520 |
| #25 | #8 AND #15 AND #24 | 501 |
|  | |  |

**Database:** AMED

**Date:** 2021-12-13

**No of results:** 102 ref

| **Search ID#** | **Search terms** | **Results** |
| --- | --- | --- |
| S23 | S6 AND S13 AND S20 - Limiters – Narrow by publication type: Academic journals,  Search modes - Find all my search terms | 102 |
| S22 | S6 AND S13 AND S20 - Limiters - Narrow by Language: - english  Search modes - Find all my search terms | 117 |
| S21 | S6 AND S13 AND S20 | 120 |
| S20 | S16 OR S19 | 91,123 |
| S19 | S17 AND S18 | 8,946 |
| S18 | TI strength OR AB strength OR KW strength | 13,788 |
| S17 | TI ( Muscle or flexor ) OR AB ( Muscle or flexor ) OR KW ( Muscle or flexor ) | 28,578 |
| S16 | S14 OR S15 | 87,341 |
| S15 | TI ( "range of motion" or recover* or outcome or function* or flexion or extension )  OR AB ( "range of motion" or recover* or outcome or function* or flexion or  extension ) OR KW ( "range of motion" or recover* or outcome or function* or  flexion or extension ) | 77,989 |
| S14 | (((ZU "treatment outcome")) or ((ZU "recovery of function"))) or  ((ZU "range of motion")) | 24,289 |
| S13 | S11 AND S12 | 238 |
| S12 | S9 OR S10 | 1,499 |
| S11 | TI ( graft or autograft or transplant* or tissue ) OR AB ( graft or autograft or  transplant* or tissue ) OR KW ( graft or autograft or transplant* or tissue ) | 10,645 |
| S10 | TI ( Hamstring or semitendinosus ) OR AB ( Hamstring or semitendinosus ) OR  KW ( Hamstring or semitendinosus ) | 1,499 |
| S9 | (ZU "hamstring muscles") | 139 |
| S8 | S6 OR S7 | 1,320 |
| S7 | (ZU "anterior cruciate ligament reconstruction") | 506 |
| S6 | S4 AND S5 | 1,320 |
| S5 | TI ( surgical* or surgery or surgeries or reconstruction* or reconstructive or  reconstructed or repair ) OR AB ( surgical* or surgery or surgeries or  reconstruction* or reconstructive or reconstructed or repair ) OR KW ( surgical*  or surgery or surgeries or reconstruction* or reconstructive or reconstructed or  repair ) | 16,201 |
| S4 | S1 OR S2 OR S3 | 2,341 |
| S3 | TI ( anterior N5 cruciate N5 ligament* ) OR AB ( anterior N5 cruciate N5 ligament* )  OR KW ( anterior N5 cruciate N5 ligament* ) | 2,280 |
| S2 | TI ACL OR AB ACL OR KW ACL | 1,105 |
| S1 | (ZU "anterior cruciate ligament") | 1,624 |
| ** TI betyder title, AB betyder abstract, ZU betyder indexerat ord, N5 betyder att det kan finnas max fem ord  mellan orden.* | | |

**Database:** PedRO

**Date:** 2021-12-13

**No of results:** 9

Anterior AND Cruciate AND Ligament AND reconstruction AND outcome = 9 träffar

**Online resource 2**

Antal unika träffar

(n = 1430)

Dubbletter
(n = 591)

Totalt
(n = 2021)

Cochrane Library

(n = 1 review

n = 179 trials)

Pedro

(n = 3)

Embase

(n = 1404)

Medline

(n = 409)

AMED

(n = 25)

**Database:** MEDLINE (OVID)

**Date:** 2022-01-12

**No of results:** 409 ref

| **#** | **Query** | **Search results** |
| --- | --- | --- |
| 1 | exp Anterior Cruciate Ligament/ | 11,560 |
| 2 | ACL.ab,kw,ti. | 18,454 |
| 3 | (anterior adj5 cruciate adj5 ligament*).ab,kw,ti. | 21,234 |
| 4 | 1 or 2 or 3 | 28,192 |
| 5 | (surgical* or surgery or surgeries or reconstruction* or reconstructive or reconstructed or repair).ab,kw,ti. | 2,440,118 |
| 6 | 4 and 5 | 17,487 |
| 7 | exp Anterior Cruciate Ligament Reconstruction/ | 6,272 |
| 8 | 6 or 7 | 17,898 |
| 9 | Hamstring Tendons/tr | 376 |
| 10 | Hamstring Muscles/tr | 65 |
| 11 | Hamstring or semitendinosus).ab,kw,ti. | 10,321 |
| 12 | (graft or autograft or transplant* or tissue).ab,kw,ti. | 2,086,500 |
| 13 | 9 or 10 | 436 |
| 14 | 11 and 12 | 3,636 |
| 15 | 13 or 14 | 3,723 |
| 16 | (re-injur* or reinjur* or rerupt*).ab,kw,ti. | 2,577 |
| 17 | (second* or Subsequen*).ab,kw,ti. limit 24 to (danish or english or norwegian or swedish) | 2,659,626 |
| 18 | exp Reinjuries/ | 82 |
| 19 | 16 or 17 or 18 | 2,661,668 |
| 20 | 8 and 15 and 19 | 466 |
| 21 | (animals not (animals and humans)).sh. | 4,908,121 |
| 22 | 20 not 21 | 456 |
| 23 | (Letter or Comment or Editorial or Case Reports).pt. | 4,044,141 |
| 24 | 22 not 23 | 430 |
| 25 | limit 24 to (danish or english or norwegian or swedish) | 409 |

**exp/** = term from the Medline controlled vocabulary, including terms found below this term in the MeSH hierarchy

**/** = term from the Medline controlled vocabulary, does not include terms found below this term in the MeSH hierarchy

**.ti,ab,kf**. = title, abstract and author keywords

**adjx** = within x words, regardless of order

***** = truncation of word for alternate endings

**Database:** EMBASE (OVID)

**Date:** 2022-01-13

**No of results:** 1403 ref

| **#** | **Search** | **Results** |
| --- | --- | --- |
| 1 | exp Anterior Cruciate Ligament/ | 12,679 |
| 2 | ACL.ab,kw,ti. | 24,116 |
| 3 | (anterior adj5 cruciate adj5 ligament*).ab,kw,ti. | 24,904 |
| 4 | 1 or 2 or 3 | 35,784 |
| 5 | (surgical* or surgery or surgeries or reconstruction* or reconstructive or reconstructed or repair).ab,kw,ti. | 3,213,024 |
| 6 | 4 and 5 | 24,500 |
| 7 | exp Anterior Cruciate Ligament Reconstruction/ | 12,769 |
| 8 | 6 or 7 | 23,059 |
| 9 | *hamstring tendon/ | 781 |
| 10 | *hamstring muscle/ | 313 |
| 11 | (Hamstring or semitendinosus).ab,kw,ti. | 12,140 |
| 12 | (graft or autograft or transplant* or tissue).ab,kw,ti. | 2,776,860 |
| 13 | 9 or 10 or 11 | 12,250 |
| 14 | 12 and 13 | 4,440 |
| 15 | (re-injur* or reinjur* or rerupt*).ab,kw,ti. | 3,083 |
| 16 | (second* or Subsequen*).ab,kw,ti. | 3,537,979 |
| 17 | exp reinjuries/ | 2,370,446 |
| 18 | 15 or 16 or 17 | 5,592,946 |
| 19 | 8 and 14 and 18 | 1,851 |
| 20 | (books or conference abstract or "conference review" or editorial or erratum or letter or short survey or tombstone).pt. | 6,837,529 |
| 21 | 19 not 20 | 1,560 |
| 22 | (animal not (animal and human)).sh. | 1,131,690 |
| 23 | 21 not 22 | 1,539 |
| 24 | limit 23 to (danish or english or norwegian or swedish) | 1,404 |

**exp/** = term from the Medline controlled vocabulary, including terms found below this term in the MeSH hierarchy

**/** = term from the Medline controlled vocabulary, does not include terms found below this term in the MeSH hierarchy

**.ti,ab,kf**. = title, abstract and author keywords

**adjx** = within x words, regardless of order

***** = truncation of word for alternate endings

**Database:** The Cochrane Library
**Date:** 2022-01-14
**No of results:** 180 ref

*Cochrane reviews: 1
Cochrane protocols: 0
Trials: 179
Editorials: 0
Special collections: 0
Clinical answers: 0*

| **ID** | **Search** | **Hits** |
| --- | --- | --- |
| #1 | MeSH descriptor: [Anterior Cruciate Ligament] explode all trees | 671 |
| #2 | (ACL):ti,ab,kw | 2,171 |
| #3 | ("anterior cruciate ligament"):ti,ab,kw | 2,862 |
| #4 | #1 OR #2 OR #3 | 3,315 |
| #5 | (surgical* or surgery or surgeries or reconstruction* or reconstructive or reconstructed or repair):ti,ab,kw | 275,547 |
| #6 | #4 AND #5 | 2,718 |
| #7 | MeSH descriptor: [Anterior Cruciate Ligament Reconstruction] explode all trees | 475 |
| #8 | #6 OR #7 | 2,719 |
| #9 | MeSH descriptor: [Hamstring Tendons] explode all trees and with  qualifier(s): [transplantation – TR] | 38 |
| #10 | MeSH descriptor: [Hamstring Muscles] explode all trees and with qualifier(s): [transplantation - TR] | 8 |
| #11 | #9 OR #10 | 44 |
| #12 | (Hamstring or semitendinosus):ti,ab,kw | 2,445 |
| #13 | (graft or autograft or transplant* or tissue):ti,ab,kw | 152,919 |
| #14 | #12 AND #13 | 795 |
| #15 | #11 OR #14 | 795 |
| #16 | MeSH descriptor: [Reinjuries] explode all trees | 4 |
| #17 | (re-injur* or reinjur* or rerupt*):ti,ab,kw | 428 |
| #18 | (second* or Subsequen*):ti,ab,kw | 430,800 |
| #19 | #16 OR #17 OR #18 | 431,041 |
| #20 | #8 AND #15 AND #19 | 180 |

**Database:** AMED

**Date:** 2022-01-14

**No of results:** 25 ref

| **Search ID#** | **Search terms** | **Results** |
| --- | --- | --- |
| S20 | S6 AND S13 AND S20 - Limiters – Narrow by publication type: Academic journals,  Search modes - Find all my search terms | 22 |
| S19 | S8 AND S13 AND S20 - Limiters - Narrow by Language: - english  Search modes - Find all my search terms | 25 |
| S18 | S8 AND S13 AND S17 | 25 |
| S17 | S14 OR S15 OR S16 | 20,115 |
| S16 | TI (second* or Subsequen*) OR AB (second* or Subsequen*) OR KW (second* or Subsequen*) | 19,868 |
| S15 | TI (re-injur* or reinjur* orrerupt*) OR AB (re-injur*or reinjur* or rerupt*) OR KW  (re-injur* or reinjur*or rerupt*) | 307 |
| S14 | (ZU "reinjuries") | 0 |
| S13 | S11 AND S12 | 238 |
| S12 | S9 OR S10 | 1,504 |
| S11 | TI ( graft or autograft or transplant* or tissue ) OR AB ( graft or autograft or  transplant* or tissue ) OR KW ( graft or autograft or transplant* or tissue ) | 10,709 |
| S10 | TI ( Hamstring or semitendinosus ) OR AB ( Hamstring or semitendinosus ) OR  KW ( Hamstring or semitendinosus ) | 1,504 |
| S9 | (ZU "hamstring muscles") | 142 |
| S8 | S6 OR S7 | 1,327 |
| S7 | (ZU "anterior cruciate ligament reconstruction") | 511 |
| S6 | S4 AND S5 | 1,327 |
| S5 | TI ( surgical* or surgery or surgeries or reconstruction* or reconstructive or  reconstructed or repair ) OR AB ( surgical* or surgery or surgeries or  reconstruction* or reconstructive or reconstructed or repair ) OR KW ( surgical*  or surgery or surgeries or reconstruction* or reconstructive or reconstructed or  repair ) | 16,258 |
| S4 | S1 OR S2 OR S3 | 2,349 |
| S3 | TI ( anterior N5 cruciate N5 ligament* ) OR AB ( anterior N5 cruciate N5 ligament* )  OR KW ( anterior N5 cruciate N5 ligament* ) | 2,288 |
| S2 | TI ACL OR AB ACL OR KW ACL | 1,108 |
| S1 | (ZU "anterior cruciate ligament") | 1,626 |
| ** TI betyder title, AB betyder abstract, ZU betyder indexerat ord, N5 betyder att det kan finnas max fem ord  mellan orden.* | | |

**Database:** PedRO

**Date:** 2021-01-13

**No of results:** 3

Anterior AND Cruciate AND Ligament AND reconstruction AND reinjur* - 2ref

Anterior AND Cruciate AND Ligament AND reconstruction AND subsequent injur* – 1ref

**Online resource 3**

Updated search in Januari 2023.

Dubbletter*
(n = 3944)

Totalt
(n = 5073)

Antal unika träffar

(n = 1129)

Embase

(n =2962)

Cochrane Library

(n =365)

Amed

(n=100)

PEDro

(n =14)

Medline

(n =1632)

**Database:** MEDLINE (OVID)

**Date:** 2023-01-10

**No of results:** 1632 ref

| 1 | exp Anterior Cruciate Ligament/ | 12067 |
| --- | --- | --- |
| 2 | ACL.ab,kf,ti. | 20188 |
| 3 | (anterior adj5 cruciate adj5 ligament*).ab,kf,ti. | 23272 |
| 4 | 1 or 2 or 3 | 30392 |
| 5 | (surgical* or surgery or surgeries or reconstruct* or repair*).ab,kf,ti. | 2705772 |
| 6 | 4 and 5 | 19043 |
| 7 | exp Anterior Cruciate Ligament Reconstruction/ | 7084 |
| 8 | 6 or 7 | 19493 |
| 9 | Hamstring Tendons/tr | 432 |
| 10 | Hamstring Muscles/tr | 66 |
| 11 | 9 or 10 | 493 |
| 12 | Hamstring Tendons/ or Hamstring Muscles/ | 1996 |
| 13 | (Hamstring* or semitendinos*).ab,kf,ti. | 13171 |
| 14 | 12 or 13 | 13462 |
| 15 | (graft* or autograft* or transplant* or tissue).ab,kf,ti. | 2318059 |
| 16 | 14 and 15 | 4587 |
| 17 | 11 or 16 | 4614 |
| 18 | exp "Recovery of Function"/ | 58853 |
| 19 | exp "Range of Motion, Articular"/ | 59227 |
| 20 | ("range of motion" or recover* or function* or flexion or extension).ab,kf,ti. | 5130353 |
| 21 | 18 or 19 or 20 | 5160820 |
| 22 | (Muscle or flexor).ab,kf,ti. | 739289 |
| 23 | strength.ab,kf,ti. | 361494 |
| 24 | 22 and 23 | 53844 |
| 25 | 21 or 24 | 5183869 |
| 26 | 8 and 17 and 25 | 1570 |
| 27 | exp Reinjuries/ | 129 |
| 28 | (re-injur* or reinjur* or rerupt*).ab,kf,ti. | 2833 |
| 29 | (second* or Subsequen*).ab,kf,ti. | 2837104 |
| 30 | 27 or 28 or 29 | 2839344 |
| 31 | 8 and 17 and 30 | 575 |
| 32 | 26 or 31 | 1839 |
| 33 | (animals not (animals and humans)).sh. | 5046442 |
| 34 | 32 not 33 | 1804 |
| 35 | (Letter or Comment or Editorial or Case Reports).pt. | 4207028 |
| 36 | 34 not 35 | 1721 |
| 37 | limit 36 to (danish or english or norwegian or swedish) | 1632 |

**exp/** = term from the Medline controlled vocabulary, including terms found below this term in the hierarchy

**/** = term from the Medline controlled vocabulary, does not include terms found below this term in the hierarchy

**.ti,ab,kf**. = title, abstract and author keywords

**adjx** = within x words, regardless of order

***** = truncation of word for alternate endings

**Database:** EMBASE (OVID)

**Date:** 2023-01-10

**No of results:** 2962 ref

| 1 | exp anterior cruciate ligament/ | 13327 |
| --- | --- | --- |
| 2 | ACL.ab,kf,ti. | 26813 |
| 3 | (anterior adj5 cruciate adj5 ligament*).ab,kf,ti. | 27747 |
| 4 | 1 or 2 or 3 | 38911 |
| 5 | (surgical* or surgery or surgeries or reconstruct* or repair*).ab,kf,ti. | 3507927 |
| 6 | 4 and 5 | 23712 |
| 7 | exp Anterior Cruciate Ligament Reconstruction/ | 14577 |
| 8 | 6 or 7 | 25495 |
| 9 | exp hamstring tendon/ | 1256 |
| 10 | exp hamstring muscle/ | 8925 |
| 11 | (Hamstring* or semitendinos*).ab,kf,ti. | 15999 |
| 12 | 9 or 10 or 11 | 19422 |
| 13 | (graft* or autograft* or transplant* or tissue).ab,kf,ti. | 3073895 |
| 14 | 12 and 13 | 6202 |
| 15 | exp convalescence/ | 56262 |
| 16 | exp "joint characteristics and functions"/ | 104583 |
| 17 | ("range of motion" or recover* or function* or flexion or extension).ab,kf,ti. | 6372937 |
| 18 | 15 or 16 or 17 | 6433716 |
| 19 | (Muscle or flexor).ab,kf,ti. | 920300 |
| 20 | strength.ab,kf,ti. | 414438 |
| 21 | 19 and 20 | 74970 |
| 22 | 18 or 21 | 6464590 |
| 23 | 8 and 14 and 22 | 2119 |
| 24 | exp Reinjuries/ | 2518395 |
| 25 | (re-injur* or reinjur* or rerupt*).ab,kf,ti. | 3416 |
| 26 | (second* or subsequen*).ab,kf,ti. | 3787381 |
| 27 | 24 or 25 or 26 | 5965677 |
| 28 | 8 and 14 and 27 | 2542 |
| 29 | 23 or 28 | 3228 |
| 30 | animal/ not (animal/ and human/) | 1172192 |
| 31 | 29 not 30 | 3192 |
| 32 | (comment or editorial or letter).pt. | 2001518 |
| 33 | 31 not 32 | 3174 |
| 34 | limit 33 to (danish or english or norwegian or swedish) | 2962 |

**exp/** = term from the Emtree controlled vocabulary, including terms found below this term in the hierarchy

**/** = term from the Medline controlled vocabulary, does not include terms found below this term in thehierarchy

**.ti,ab,kf**. = title, abstract and author keywords

**adjx** = within x words, regardless of order

***** = truncation of word for alternate endings

**Database:** The Cochrane Library
**Date:** 2023-01-10
**No of results:** 365 ref

*Cochrane reviews: 1
Cochrane protocols: 0
Trials: 364
Editorials: 0
Special collections: 0*

| #1 | MeSH descriptor: [Anterior Cruciate Ligament] explode all trees | 688 |
| --- | --- | --- |
| #2 | (ACL):ti,ab,kw | 2411 |
| #3 | (anterior near/5 cruciate near/5 ligament*):ti,ab,kw | 3167 |
| #4 | #1 OR #2 OR #3 | 3636 |
| #5 | (surgical* or surgery or surgeries or reconstruct* or repair*):ti,ab,kw | 299578 |
| #6 | #4 AND #5 | 3001 |
| #7 | MeSH descriptor: [Anterior Cruciate Ligament Reconstruction] explode all trees | 522 |
| #8 | #6 OR #7 | 3002 |
| #9 | MeSH descriptor: [Hamstring Tendons] explode all trees | 52 |
| #10 | MeSH descriptor: [Hamstring Muscles] explode all trees | 181 |
| #11 | (Hamstring* or semitendinos*):ti,ab,kw | 3214 |
| #12 | #9 OR #10 OR #11 | 3214 |
| #13 | (graft* or autograft* or transplant* or tissue):ti,ab,kw | 168198 |
| #14 | #12 AND #13 | 984 |
| #15 | MeSH descriptor: [Recovery of Function] explode all trees | 5740 |
| #16 | MeSH descriptor: [Range of Motion, Articular] explode all trees | 5387 |
| #17 | ("range of motion" or recover* or function* or flexion or extension):ti,ab,kw | 385685 |
| #18 | #15 OR #16 OR #17 | 385695 |
| #19 | (Muscle or flexor):ti,ab,kw | 88026 |
| #20 | (strength):ti,ab,kw | 44642 |
| #21 | #19 AND #20 | 24872 |
| #22 | #18 OR #21 | 394378 |
| #23 | #8 AND #14 AND #22 | 464 |
| #24 | MeSH descriptor: [Reinjuries] explode all trees | 8 |
| #25 | (re-injur* or reinjur* or rerupt*):ti,ab,kw | 478 |
| #26 | (second* or Subsequen*):ti,ab,kw | 481203 |
| #27 | #24 OR #25 OR #26 | 481465 |
| #28 | #8 AND #14 AND #27 | 233 |
| #29 | #23 OR #28 | 551 |
| #30 | (clinicaltrials OR trialsearch):so | 443182 |
| #31 | (conference proceeding):pt | 215178 |
| #32 | #30 OR #31 | 658360 |
| #33 | #29 NOT #32 | 365 |

**MeSH descriptor: [] explode all trees:** term from the MeSH controlled vocabulary, including terms found below this term in the hierarchy

**.ti,ab,kw**. = title, abstract and author keywords

**Near/** = within x words, regardless of order

***** = truncation of word for alternate endings

**Database:** AMED

**Date:** 2023-01-10

**No of results:** 100 ref

| S28 | S22 OR S26  Narrow by Language: - english | 100 |
| --- | --- | --- |
| S27 | S22 OR S26 | 117 |
| S26 | S8 AND S13 AND S25 | 27 |
| S25 | S23 OR S24 | 21,189 |
| S24 | TI ( second* or subsequen* ) OR AB ( second* or subsequen* ) OR KW ( second* or subsequen* ) | 20,925 |
| S23 | TI ( re-injur* or reinjur* or rerupt* ) OR AB ( re-injur* or reinjur* or rerupt* ) OR KW ( re-injur* or reinjur* or rerupt* ) | 330 |
| S22 | S8 AND S13 AND S21 | 111 |
| S21 | S17 OR S20 | 64,632 |
| S20 | S18 AND S19 | 9,306 |
|  |  |  |
|  |  |  |
| S19 | TI strength OR AB strength OR KW strength | 14,379 |
|  |  |  |
|  |  |  |
| S18 | TI ( Muscle or flexor ) OR AB ( Muscle or flexor ) OR KW ( Muscle or flexor ) | 29,735 |
| S17 | S14 OR S15 OR S16 | 60,117 |
| S16 | TI ( "range of motion" or recover* or function* or flexion or extension ) OR AB ( "range of motion" or recover* or function* or flexion or extension ) OR KW ( "range of motion" or recover* or function* or flexion or extension ) | 59,748 |
| S15 | (ZU "range of motion") | 5,246 |
|  |  |  |
|  |  |  |
| S14 | (ZU "recovery of function") | 1,205 |
| S13 | S11 AND S12 | 263 |
| S12 | S9 OR S10 | 1,597 |
| S11 | TI ( graft* or autograft* or transplant* or tissue ) OR AB ( graft* or autograft* or transplant* or tissue ) OR KW ( graft* or autograft* or transplant* or tissue ) | 11,538 |
| S10 | TI ( Hamstring* or semitendinos* ) OR AB ( Hamstring* or semitendinos* ) OR KW ( Hamstring* or semitendinos* ) | 1,597 |
| S9 | (ZU "hamstring muscles") | 177 |
| S8 | S6 OR S7 | 1,421 |
|  |  |  |
|  |  |  |
| S7 | (ZU "anterior cruciate ligament reconstruction") | 593 |
| S6 | S4 AND S5 | 1,421 |
| S5 | TI ( surgical* or surgery or surgeries or reconstruct* or repair* ) OR AB ( surgical* or surgery or surgeries or reconstruct* or repair* ) OR KW ( surgical* or surgery or surgeries or reconstruct* or repair* ) | 17,288 |
| S4 | S1 OR S2 OR S3 | 2,476 |
| S3 | TI ( anterior N5 cruciate N5 ligament* ) OR AB ( anterior N5 cruciate N5 ligament* ) OR KW ( anterior N5 cruciate N5 ligament* ) | 2,413 |
| S2 | TI ACL OR AB ACL OR KW ACL | 1,174 |
| S1 | (ZU "anterior cruciate ligament") | 1,672 |

**ZU:** term from the Amed controlled vocabulary, including terms found below this term in the hierarchy

**.ti,ab,kw**. = title, abstract and author keywords

**Nx** = within x words, regardless of order

***** = truncation of word for alternate endings

**Database:** PedRO

**Date:** 2023-01-10

**No of results:** 14

Pedro 1: Anterior AND Cruciate AND Ligament AND reconstruct* AND reinjur* = 3ref

Pedro 2: Anterior AND Cruciate AND Ligament AND reconstruct* AND subsequent injur* = 2 ref

Pedro 3: Anterior AND Cruciate AND Ligament AND reconstruction AND outcome = 9ref

***** = truncation of word for alternate endings
