# Supplementary material for: Restoring Knee Flexor Strength Symmetry Requires 2 Years After ACL Reconstruction, But Does It Matter for Second ACL Injuries? A Systematic Review and Meta-analysis
Source: Sports Med Open. 2024 Jan 5;10:2. doi: 10.1186/s40798-023-00666-5 (PMC10769975; doi:10.1186/s40798-023-00666-5)
Supplement: Supplementary file 2 — Additional file 2. Complementary Result Tables. [file 40798_2023_666_MOESM2_ESM.docx]

**Appendix**

*Appendix Table S1. Recovery of knee flexor strength symmetry at velocities other than 60°/s and 180°/s and/or at timepoints other than preoperatively and 3, 6, 12 and 24 months in studies not included in the meta-analysis.*

| Studies | Subgroups  (e.g graft choice) | Angular velocity | Timepoint of assessment (months) | Limb symmetry index (mean + SD) |
| --- | --- | --- | --- | --- |
| Barenius et al. 2013 [27] | STG | 60°/s | 37 ± 6 | 78.0% ± 36% |
|  | ST |  | 36 ± 4 | 85.8% ± 30% |
| Beaudoin et al. 2022 [28] | Ipsilateral graft, seated | 90°/s | 153.6 ± 20.4 | 94.6% ± 16.4% |
|  | Ipsilateral graft, supine |  |  | 87.8% ± 18.7% |
| Carter et al. 1999 [30] | ST | 300°/s | 6 (6-7) | 79.1% ± 27.4% |
|  | STG |  |  | 75.6% ± 19.0% |
| Chen et al. 2010 [32] | NA | 60°/s, 90°/s and 180°/s | Preoperative | 80.2% ± 11.7%  (Mean of 60-180°/s) |
|  |  |  | 55 (48-84) | 90.2% ± 11.2%  (Mean of 60-180°/s) |
| Chen et al. 2004 [33] | NA | 60°/s, 90°/s and 180°/s | Preoperative | 83.3% ± 9.0%  (Mean of 60-180°/s) |
|  |  |  | 28 (24-38) | 94.0% ± 9.5%  (Mean of 60-180°/s) |
| Cristiani et al. 2019 [34] | HT group | 90°/s | 6 | 89.3% ± 15.3% |
| Ebert et al. 2019 [35] | NA | 90°/s | 12 | 93.0% ± 14.7% |
|  |  |  | 24 | 96.8% ± 15.4% |
| Ebert et al. 2021 [36] | DB HT | 90°/s | 92.4 ± 7.2 (84-114) | 97.6% ± 11.5% |
|  | DB HT + LARS |  | 94.8 ± 10.8 (84-120) | 95.1% ± 11.6% |
| Ebert et al. 2021 [37] | NA | 90°/s | 10.8 ± 1.4 (9-12) | 97.6% ± 5.5% |
| Ebert et al. 2022 [38] | Accelerated | 90°/s | 6 | 96.7% ± 11.6% |
|  | Control |  | 9 | 96.3% ± 13.4% |
|  |  |  | 12 | 97.8% ± 8.7% |
|  |  |  | 24 | 96.3% ± 7.6% |
|  |  |  | 6 | 93.5% ± 10.5% |
|  |  |  | 9 | 91.1% ± 10.5% |
|  |  |  | 12 | 94.0% ± 12.3% |
|  |  |  | 24 | 93.7% ± 10.5% |
| Fischer et al. 2018 [40] | NA | 60°/s | 5.4 ± 1.3 (3-9.4) | 86.3% ± 14.6% |
|  |  |  | 7.5 ± 1.8 (4.8-15) | 90.1% ± 13.1% |
| Harilainen et al. 2006 [46] | HT group | 60°/s | 60 (47-79) | 97.5% ± 12.1% |
|  |  | 180°/s |  | 96.5% ± 12.7% |
| Harput et al. 2018 [47] | NA | 90°/s | 6 | 95.3% ± 14.6% |
| Holm et al. 2010 [49] | HT group | 60°/s | 120 | 98.0% ± 28.5% |
|  |  | 240°/s |  | 92.6% ± 25.7% |
| Högberg et al. 2022 [50] | NA | 90°/s | 2.5 | 88.3% ± 15% |
|  |  |  | 4 | 94.0% ± 14% |
|  |  |  | 6 | 94.1% ± 11% |
|  |  |  | 12 | 95.2% ± 9% |
| Iriuchishima et al. 2010 [52] | Standard | 60°/s | 9 | 91% ± 12.4% |
|  | Accelerated |  |  | 87.7% ± 11.4% |
| Karlson et al. 1994 [54] | OTT | 60°/s | 39.6 ± 8.4 | 102% ± 20% |
|  | TCC |  | 28.8 ± 3.6 | 98% ± 15% |
| Kılınç et al. 2015 [55] | NA | 60°/s | 23.1 ± 9.1 (9-42) | 93.6% ± 14.7% |
|  |  | 240°/s |  | 93.3% ± 31.0% |
| Koga et al. 2015 [57] | SB HT | 60°/s | 71 (36-140) | 86.6% ± 14.3% |
|  | DB HT |  | 68 (36-136) | 94.2% ± 11.6% |
| Kondo et al. 2016 [59] | 40° tension | 60°/s | 29 (24-72) | 95.8% ± 15.3% |
|  | 30° tension |  |  | 94.1% ± 11.9% |
| Królikowska et al. 2018 [63] | Supervised 3 months | 60°/s | 7.8 ± 2.1 | 87.5% ± 17.5% |
|  |  | 180°/s |  | 87.8% ± 16.4% |
| Lautamies et al. 2008 [64] | HT group | 60°/s | 60 | 96.5% ± 12.9% |
|  |  | 180°/s |  | 95.0% ± 12.3% |
| Lesevic et al. 2020 [67] | HT group | 90°/s | 6 | 82.9% ± 33.3% |
| Maeda et al. 1996 [68] | NA | 60°/s | 27 (24-42) | 95.4% ± 16.2% |
|  |  | 180°/s |  | 96.6% ± 17.6% |
| Matsumoto et al. 2006 [69] | HT group | 60°/s | 80.7 ± 13.2 | 85.6% ± 13.1% |
|  |  | 180°/s |  | 91.4% ± 13.5% |
| Ogborn et al. 2021 [73] | Seated | 90°/s | 168 ± 52.8 | 95.8% ± 17.5% |
|  | Supine |  |  | 93.8% ± 13.9% |
| Sanada et al. 2021 [76] | HT group | 60°/s | 8 | 85.6% ± 11.9% |
|  |  | 180°/s |  | 93.5% ± 13.3% |
| Tim-Yun Ong et al. 2022 [85] | NA | 60°/s | 6-9 | 89.1% ± 11% |
|  |  | 180°/s |  | 96.6% ± 12% |
| Tsuda et al. 2009 [86] | Women | 60°/s | 18 | 100% ± 14% |
|  |  |  | 37 (25-47) | 96% ± 10% |
|  | Men |  | 18 | 92% ± 7% |
|  |  |  | 37 (25-47) | 95% ± 7% |
| Witvrouw et al. 2001 [88] | HT group | 240°/s | Preoperative | 88.6% ± 15.3% |
|  |  |  | 6 | 82.2% ± 18.9% |
|  |  |  | 12 | 85.8% ± 14.9% |

*DB = Double-Bundle; HT = Hamstring tendon autograft, unspecified; LARS = Ligament Augmentation and Reconstruction System; NA = Not applicable; OTT = Over-the-top graft placement; SB = Single-Bundle; ST = Semitendinosus alone; STG = Semitendinosus + gracilis; TCC = Through the condyle graft placement*

*Appendix Table S2. Methodology for measuring knee flexor strength symmetry*

| Studies | Test apparatus | Contraction type | Position | Range of motion | Velocity | Repetitions | Set-rest (s) |
| --- | --- | --- | --- | --- | --- | --- | --- |
| Araki et al. 2011 [25] | Biodex | Missing | Missing | Missing | 60°/s | Missing | Missing |
| Baba et al. 2019 [26] | Cybex II | Missing | Missing | Missing | 60°/s | Missing | Missing |
| Barenius et al. 2013 [27] | Biodex | Missing | Seated | Missing | 60°/s | Missing | Missing |
| Beaudoin et al. 2022 [28] | Biodex 3 | Concentric | Seated, Supine | 0-95° | 90°/s | 5 | Missing |
| Blucher et al. 2022 [29] | Humac Norm | Missing | Seated | Missing | 60°/s, 180°/s | 3, 5 | Missing |
| Carter et al. 1999 [30] | Cybex II | Missing | Seated | Missing | 180°/s, 300°/s | 5, 15 | Missing |
| Chantrelle et al. 2023 [31] | Humac | Missing | Seated | 0-100° | 60°/s, 180°/s | 3, 5 | 30 |
| Chen et al. 2010 [32] | Cybex 340 | Missing | Missing | Missing | 60°/s, 90°/s, 180°/s | Missing | Missing |
| Chen et al. 2004 [33] | Cybex 340 | Missing | Missing | Missing | 60°/s, 90°/s, 180°/s | Missing | Missing |
| Cristiani et al. 2019 [34] | Biodex 3 | Missing | Missing | 10 - 90° | 90°/s | 5 | Missing |
| Ebert et al. 2019 [35] | Isosport | Concentric | Missing | Missing | 90°/s | Missing | Missing |
| Ebert et al. 2021 [36] | Isosport | Missing | Missing | Missing | 90°/s | Missing | Missing |
| Ebert et al. 2021 [37] | Isosport | Concentric | Seated | Missing | 90°/s | 1 | Missing |
| Ebert et al. 2022 [38] | Isosport | Missing | Missing | Missing | 90°/s | Missing | Missing |
| Fabbriciani et al. 2005 [39] | Genu Plus II | Missing | Missing | Missing | 180°/s | 15 | Missing |
| Fischer et al. 2018 [40] | Con-Trex | Concentric | Missing | Missing | 60°/s | 4 | 120 |
| Gifstad et al. 2013 [41] | Biodex | Missing | Missing | Missing | 60°/s | Missing | Missing |
| Guney-Deniz et al. 2020 [42] | Biodex 3 | Concentric | Seated | 0-90° | 60°/s, 180°/s | 10 | 120 |
| Hamada et al. 2001 [43] | Cybex 6000 | Missing | Missing | Missing | 60°/s, 180°/s | Missing | Missing |
| Hanada et al. 2019 [44] | Biodex | Missing | Missing | Missing | 60°/s | Missing | Missing |
| Harilainen et al. 2005 [45] | Lido Multijoint II | Missing | Missing | Missing | 60°/s, 180°/s | Missing | Missing |
| Harilainen et al. 2006 [46] | Lido Multijoint II | Missing | Missing | 0-80° | 60°/s, 180°/s | Missing | Missing |
| Harput et al. 2018 [47] | IsoMed 2000 | Concentric, eccentric | Seated | 0-90° | 90°/s | 5 | 120 |
| Hasebe et al. 2005 [48] | Cybex II | Missing | Missing | Missing | 60°/s | 2 | Missing |
| Holm et al. 2010 [49] | Cybex 6000 | Missing | Missing | Missing | 60°/s, 240°/s | Missing | Missing |
| Högberg et al. 2022 [50] | Biodex 4 | Concentric | Seated | 0-90° | 90°/s | 1 | 30 |
| Inagaki et al. 2013 [51] | Kin-Com | Missing | Missing | Missing | 60°/s, | Missing | Missing |
| Iriuchishima et al. 2010 [52] | Cybex II | Missing | Seated | 0-100° | 60°/s | 5 | Missing |
| Johnston et al. 2022 [53] | Humac Norm | Concentric | Seated | Missing | 60°/s, 180°/s | 3, 5 | Missing |
| Karlson et al. 1994 [54] | Orthotron | Missing | Missing | Missing | 60°/s | 3 | Missing |
| Kılınç et al. 2015 [55] | Cybex II | Concentric | Missing | 0-90° | 60°/s, 240°/s | 5 | 60 |
| Koga et al. 2015 [56] | Cybex | Missing | Missing | Missing | 60°/s | Missing | Missing |
| Koga et al. 2015 [57] | Cybex | Missing | Missing | Missing | 60°/s | Missing | Missing |
| Kondo et al. 2012 [58] | Cybex II | Missing | Missing | Missing | 60°/s | Missing | Missing |
| Kondo et al. 2016 [59] | Cybex II | Missing | Missing | Missing | 60°/s | Missing | Missing |
| Kouloumentas et al. 2019 [60] | Biodex 4 | Missing | Missing | Missing | 60°/s, 180°/s | Missing | Missing |
| Koutras et al. 2013 [10] | Missing | Missing | Missing | Missing | 60°/s, 180°/s | 3 | Missing |
| Królikowska et al. 2019 [62] | Humac Norm | Concentric | Seated | Missing | 180°/s | 10 | 120 |
| Królikowska et al. 2018 [63] | Humac Norm | Concentric | Seated | Missing | 60°/s, 180°/s | 5, 10 | 120 |
| Lautamies et al. 2008 [64] | Lido Multijoint II | Concentric | Seated | 0-80° | 60°/s,  180°/s | 5 | 60 |
| Lee et al. 2015 [65] | Biodex 4 | Concentric | Seated | 0-100° | 60°/s | 5 | 30 |
| Lee et al. 2016 [66] | Cybex II | Missing | Missing | Missing | 60°/s, 180°/s | Missing | Missing |
| Lesevic et al. 2020 [67] | Biodex 4 | Concentric | Missing | Missing | 90°/s | 8 | Missing |
| Maeda et al. 1996 [68] | Cybex II | Missing | Missing | Missing | 60°/s, 180°/s | Missing | Missing |
| Matsumoto et al. 2006 [69] | Cybex II | Missing | Missing | Missing | 60°/s, 180°/s | Missing | Missing |
| Murray et al. 2019 [70] | Biodex 3 | Missing | Missing | Missing | 60°/s | Missing | Missing |
| Nakamura et al. 2002 [71] | Cybex II | Missing | Missing | Missing | 60°/s, 180°/s, | 3 | Missing |
| Nishio et al. 2018 [72] | Cybex II | Missing | Missing | Missing | 60°/s | 3 | Missing |
| Ogborn et al. 2021 [73] | Biodex 3 | Concentric | Seated, supine | 5-90° | 90°/s | 5 | 60 |
| Riesterer et al. 2020 [74] | Humac Norm | Concentric | Seated | 0-100° | 60°/s | 5 | 60 |
| Roman et al. 2021 [75] | Humac CSMI | Missing | Seated | 0-90° | 60°/s | 5 | Missing |
| Sanada et al. 2021 [76] | Biodex 3 | Missing | Missing | Missing | 60°/s, 180°/s | Missing | Missing |
| San Jose et al. 2022 [77] | Humac Norm | Concentric | Seated | 0-90° | 60°/s, 180°/s | 5 | 30 |
| Sengoku et al. 2022 [90] | Biodex 4 | Missing | Missing | 0-100° | 60°/s, 180°/s | 3 | Missing |
| Severyns et al. 2022 [79] | Con-Trex | Missing | Seated | Missing | 60°/s, 180°/s | Missing | Missing |
| Sinding et al. 2020 [80] | Humac Norm | Concentric, eccentric | Missing | 5-100° | 60°/s, 180°/s | 5 | Missing |
| Suh et al. 2021 [81] | Biodex 4 | Concentric | Seated | 0-100° | 180°/s | 5 | Missing |
| Tajima et al. 2021 [82] | Biodex 4 | Missing | Missing | Missing | 60°/s | Missing | Missing |
| Taketomi et al. 2018 [83] | Cybex | Concentric | Missing | Missing | 60°/s | Missing | Missing |
| Tanaka et al. 2010 [84] | Cybex II | Missing | Missing | Missing | 60°/s | Missing | Missing |
| Tim-Yun Ong et al. 2022 [85] | Biodex 4 | Concentric | Seated | Missing | 60°/s, 180°/s | 5, 10 | Missing |
| Tsuda et al. 2009 [86] | Cybex 6000 | Concentric | Missing | Missing | 60°/s | Missing | Missing |
| Ueda et al. 2021 [87] | Genu Plus | Missing | Missing | Missing | 60°/s | 5 | Missing |
| Witvrouw et al. 2001 [88] | Cybex 350 | Missing | Missing | Missing | 60°/s, 240°/s | 5 | Missing |

*S = Seconds*

*Appendix Table S3. Methodology for measuring knee flexor strength in studies presenting second ACL injuries.*

| Studies | Test apparatus | Contraction mode | Position | Range of motion | Angular velocity | Repetitions | Rest in between (s) | Timepoint for assessment, mean ± SD, range (months) |
| --- | --- | --- | --- | --- | --- | --- | --- | --- |
| Blucher et al. 2022 [29] | Humac Norm | Missing | Seated | Missing | 60°/s, 180°/s | 3, 5 | Missing | 12 |
| Severyns et al. 2022 [79] | Con-Trex | Missing | Seated | Missing | 60°/s, 180°/s | Missing | Missing | 6 |
| Tanaka, 2010 [84] | Cybex II | Missing | Missing | Missing | 60°/s | Missing | Missing | Preoperative, 6 |
| Yamanashi, 2019 [91] | Biodex System 3 | Concentric | Missing | Missing | 60°/s | Missing | Missing | 3, 6 |

*N = Numbers; NA = Not applicable; S = Seconds; SD = Standard deviation.*
